# REVISION OF THE BRIEF INTERNATIONAL CLASSIFICATION OF FUNCTIONING, DISABILITY AND HEALTH CORE SET FOR MULTIPLE SCLEROSIS: A STUDY OF THE COMPREHENSIVE ICF CORE SET FOR MULTIPLE SCLEROSIS WITH PARTICIPANTS REFERRED FOR WORK ABILITY ASSESSMENT

**DOI:** 10.2340/jrm.v56.19671

**Published:** 2024-03-07

**Authors:** Daiva VALADKEVIČIENĖ, Dalius JATUŽIS, Irena ŽUKAUSKAITĖ, Virginija DANYLAITĖ KARRENBAUER, Indre BILEVICIUTE-LJUNGAR

**Affiliations:** 1 Clinic of Neurology and Neurosurgery, Institute of Clinical Medicine, Faculty of Medicine, Vilnius University; 2 Disability and Working Capacity Assessment Office under the Ministry of Social Security and Labour of the Republic of Lithuania; 3 Institute of Psychology, Faculty of Philosophy, Vilnius University, Vilnius, Lithuania; 4 Department of Clinical Neuroscience, Karolinska Institutet; 5 Medical Unit Neuro, Karolinska University Hospital; 6 Department of Clinical Sciences, Karolinska Institutet, Stockholm, Sweden; 7 Multidisciplinary Pain Clinic, St Göran Hospital, Stockholm, Sweden

**Keywords:** multiple sclerosis, International Classification of Functioning, Disability and Health, Comprehensive ICF Core Set for MS, Brief ICF Core Set for MS, disability, ICF categories

## Abstract

**Objective:**

To evaluate the Comprehensive International Classification of Functioning, Disability and Health (ICF) Core Set for multiple sclerosis with regard to the Brief ICF Core Set for multiple sclerosis.

**Design:**

Descriptive cross-sectional single-centre study.

**Subjects:**

A total of 151 participants (99 females/52 males, mean age 49 years) referred for work ability assessment.

**Methods:**

Data were collected from clinical recordings and by telephone interview.

**Results:**

Among 33 Body Functions, 14 were impaired in over 60% of the participants, and 6 in over 75%. These 6 most impaired functions were related to exercise tolerance (b455), urination (b620), muscle power (b730), motor reflex (b750), control of voluntary movement (b760) and gait pattern (b770). Among 54 Activities and Participation categories, 8 were impaired in over 60% of the participants, and 3 were impaired in over 75%. The latter activities were related to walking (d450), moving around (d455) and moving around using equipment (d465). Among the 36 Environmental categories, most were facilitators, except for temperature (e2250) and employment (e590). The latter category was both a facilitator and a barrier.

**Conclusion:**

These results suggest additional categories that should be included into the Brief ICF Core Set, to improve its representation of the complex disability of multiple sclerosis.

LAY ABSTRACT

Multiple sclerosis induces the destruction of pathways in the central nervous system. However, the overall picture of most impaired Body Functions, Activities and Participation, and Environmental Factors, through use of the Comprehensive International Classification of Functioning, Disability and Health Core Set for multiple sclerosis, has not been reported. This study presents the results of the Comprehensive International Classification of Functioning, Disability and Health Core Set for multiple sclerosis, showing that the most impaired categories are also presented in the Brief Core Set for multiple sclerosis. In addition, several categories from the Comprehensive International Classification of Functioning, Disability and Health Core Set for multiple sclerosis were impaired in a major part of participants. The results suggest that these categories should be included in the Brief International Classification of Functioning, Disability and Health Core Set in order to cover the complex disability of multiple sclerosis from a clinician’s perspective.

Multiple sclerosis (MS) is an immune-mediated demyelinating and neurodegenerative disorder of the central nervous system that leads to impaired body functions, reduced activity and restricted participation. One way to assess the biopsychosocial model of MS is to use the World Health Organization (WHO) International Classification of Functioning, Disability and Health (ICF), which includes assessments of Body Functioning, Activity and Participation, and Environmental Factors. The ICF was officially endorsed by all 191 WHO Member States at the 54th World Health Assembly on 22 May 2001 (resolution WHO 54.21) (1) and can be used for clinical practice and research. ICF Core Sets have been developed for several neurological conditions, including MS (2, 3) and stroke (4) in 2004, and for adults with cerebral palsy in 2022 (5). A generic list of ICF categories has also been identified to capture the functional features of neurological disorder patients, mainly those with cerebrovascular diseases and head trauma (6). An online resource (https://www.icf-core-sets.org/en/page1.php) is available to create clinical documentation tools using either Brief or Comprehensive ICF Core Sets for the following neurological conditions for adults: stroke, MS, traumatic brain injury, cerebral palsy, spinal cord injury, neurological acute and post-acute conditions. A comparative study using the ICF was conducted in an Italian cohort evaluating patients with myasthenia gravis, Parkinson’s disease, and migraine (7). Another comparative study on patient-reported disability was conducted using ICF categories in an Australian cohort of patients with motor neurone disease, Guillain‒Barré syndrome, and MS (8). MS patient-reported disability and environmental factors have been described using ICF by another Australian group (9). Researchers in Finland described the evaluation of MS patients’ walking ability deterioration (10) and Activities and Participation categories (10) according to the ICF. Comprehensive ICF Core Set validation studies for MS have also been conducted from the perspective of physiotherapists (11), occupational therapists (12), language therapists (13), and neurologists (14), using the 3-round Delphi technique.

Recently, we reported results from a study using the Brief ICF Core Set for MS, which includes 20 categories of Body Functions, Activities and Participation, and Environmental factors. The results showed that the ICF category “moving around (d445)” was the most impaired activity, found in 75% of 72 participants (51% of participants had severe impairments) (15). However, the Comprehensive ICF Core Set for MS has not yet been used and validated by a physician, and studies evaluating the completeness of the Brief ICF Core Set for MS in clinical practice are lacking.

The aims of the current study were: (*i*) to practice the Comprehensive ICF Core Set for MS from the perspective of a physician; and (*ii*) to identify the most impaired Body Functions, Activities and Participation, and Environmental Factors that could be included in the Brief ICF Core Set for MS.

The study hypothesis was that previously found impairments in Body Functions, Activities and Participation, and Environmental factors will remain impaired when using the Comprehensive ICF core set for MS.

## MATERIALS AND METHODS

This cross-sectional study was conducted at Vilnius University, Faculty of Medicine, Institute of Clinical Medicine, Clinic of Neurology and Neurosurgery and at the Disability and Working Capacity Assessment Office (DWCAO) under the Ministry of Social Security and Labour of the Republic of Lithuania, Vilnius, Lithuania. Data were collected from March to August 2022.

### Participants

**Fig. 1:**
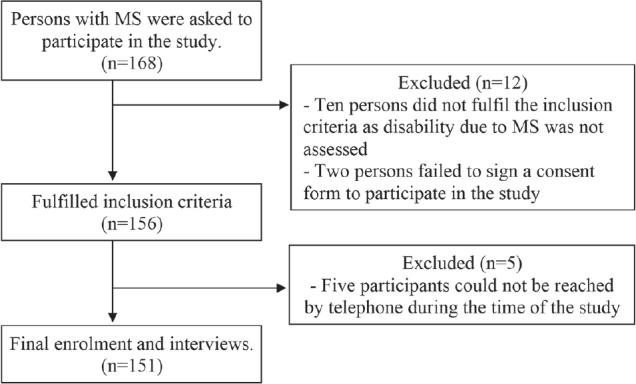
Flow chart of the study cohort selection process. MS: multiple sclerosis

A total of 151 consecutive participants with MS participated in the study. The flowchart of the study cohort selection process is presented in Fig. 1.

The inclusion criteria were as follows: (*i*) age over 18 years, but not higher than retirement age; (*ii*) MS diagnosis according to the McDonald criteria 2017 revisions (16); (*iii*) MS at remission stage and stable neurological condition; (*iv*) fluent in the Lithuanian language; (*v*) disability assessed at DWCAO; and (*vi*) voluntary consent to participate in the study certified by the participant’s signed informed consent form. The retirement age was 64 years and 4 months for men and 63 years and 8 months for women during the study period (2022).

The exclusion criteria were as follows: patients not compliant with the study protocol (reasons for exclusion are shown in Fig. 1).

### Data collection

Sociodemographic (age and sex) and clinical data (type of MS, disease duration, comorbidities and medication) were collected by the main investigator (DV) from journal recordings at DWCAO.

Comprehensive ICF data for the MS participants were collected by the main investigator (DV) by telephone interviews due to legal limitations on face-to-face contact during the SARS-CoV-2 (COVID-19) pandemic restrictions.

The duration of the telephone interview was 30–60 min, some participants requiring more than 1 call, which could extend the time up to 120 min in specific cases. During a telephone call, participants were asked if they continued to agree to participate and could answer the questions. If this was not the case, a new time was appointed. Before asking the ICF-related questions, a participant was informed about the principles of coding the ICF functions from 0 to 4, i.e. from no impairments in body functions and activities, to being dependent on assistive devices and support from other people. Questions were asked by following the ICF Comprehensive Core Set and using a coded paper sheet for marking impairments. Additional information was extracted from the journal recordings available from DWCAO regarding the following categories: b525 defecation functions, b620 urination functions, b730 muscle power functions, b735 muscle tone functions, b740 muscle endurance functions, b750 motor reflex functions, b760 control of voluntary movement functions, b770 gait pattern functions, s110 structure of brain, s120 spinal cord and related structures, drug prescriptions and aids to improve functions and disability.

The Comprehensive ICF Core Set for MS consists of 138 categories: Body Functions, 40; Body Structures, 7; Activities and Participation, 53; and Environmental Factors, 38. The following b-categories were not evaluated due to lack of available information: b1308 (energy and drive functions, other specified); b235 (vestibular functions); b445 (respiratory muscle functions); b5508 (thermoregulatory functions, other specified (sensitivity to heat)); b5508 (thermoregulatory functions, other specified (sensitivity to cold)); b640 (sexual functions), and b710 (mobility of joint functions). A detailed description of how ICF categories were assessed is presented in Appendix S1.

The following body structures were not evaluated, due to a lack of information and the inability to perform a physical assessment: s610 (structure of urinary system); s730 (structure of upper extremity); s750 (structure of lower extremity); s760 (structure of trunk) and s810 (structure of areas of skin). Among e-categories, e1108 (products or substances for personal consumption, other specified) was not evaluated. Not evaluated categories are presented in Table SI.

The Brief ICF Core Set for MS comprises 8 b-, 2 s-, 6 d- and 4 e-categories, evaluated at the second level of ICF classification of categories. Some categories in the current study were evaluated at the third level (Appendix S1). The third-level categories were transformed to the second level by taking the most impaired level of 1 of the categories.

The study was conducted after contract number (5.74) SU-2990, between Vilnius University and DWCAO, was signed on 17 November 2021. DWCAO agreed to provide personal data for the study, and Vilnius University agreed to analyse depersonalized data and ensure ethical research standards. The study was approved by the Lithuanian Bioethics Committee (No. 2021/10–1387-855), Vilnius, Lithuania. Each participant signed informed consent documentation and an agreement regarding personal data usage.

### Statistical analysis

Statistical analyses were performed using the statistical software package SPSS 17.0 (version for MS Windows). Descriptive statistics for the quantitative variables are presented as the mean and standard deviation (SD); discrete variables are presented as the absolute value and the percentage of the analysed sample group.

## RESULTS

### Study cohort

A total of 151 participants referred to DWCAO fulfilled the inclusion criteria. The flowchart of the included patients and reasons for drop-out/exclusion are shown in Fig. 1.

Details of the sociodemographic and clinical variables are presented in Table I.

**Table I:** Clinical and sociodemographic characteristics

Variables	Full sample (*N* = 151)
Age, years, mean ± SD	49.3 ± 10.5
Time from symptoms, years, mean ± SD	13.6 ± 9.1
Time from diagnosis, years, mean ± SD	11.3 ± 8.0
EDSS score years, mean ± SD	4.6 ± 1.3
Sex: male, *n* (%)	52 (34.4)
Education, *n* (%)	
Basic	7 (4.6)
Secondary	16 (10.6)
Vocational	40 (26.5)
College	30 (19.9)
Higher	58 (38.4)
Employment: active, *n* (%)	71 (47.0)
Type of disease, *n* (%)	
SPMS	19 (12.6)
PPMS	11 (7.3)
RRMS	121 (80.1)
DMT, *n* (%)	
Moderate efficacy	66 (43.7)
High efficacy	57 (37.7)
Untreated	28 (18.5)
Comorbidities, *n* (%)	
None	112 (74.2)
One	33 (21.9)
Two	6 (4.0)

DMT: disease-modifying treatments; EDSS: Expanded Disability Status Scale; SD: standard deviation; SPMS: secondary-progressive multiple sclerosis; PPMS: primary-progressive multiple sclerosis; RRMS: relapsing-remitting multiple sclerosis.

Briefly, a cohort with a mean age of 49.3 years and a mean Expanded Disability Status Scale (EDSS) score of 4.6 included 65% females, and 80% of the participants were diagnosed with relapsing-remitting MS. A high proportion of patients were undergoing immunomodulatory treatment (43.7% were taking moderate-efficacy treatment and 37.7 high-efficacy treatment), and 18.5% were untreated. Most of the patients (74.2%) were free from comorbidities, and 25.9% had secondary diagnoses, as summarized in Table SII.

### Comprehensive ICF Core Set for multiple sclerosis

**Fig. 2:**
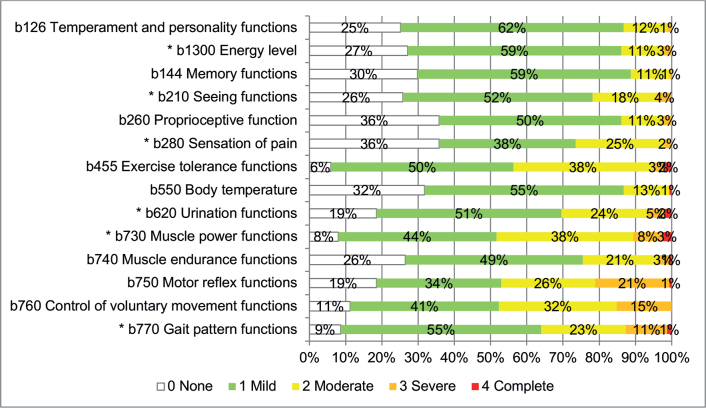
Impairments in Body Functions according to the Comprehensive International Classification of Functioning, Disability and Health (ICF) Core Set for multiple sclerosis (MS) (*N* = 151) in over 60% of the participants are presented in percentage points. *b-categories included in the Brief ICF Core Set for MS.

*Body Functions*. Among the 33 Body Functions categories evaluated, 14 were impaired in over 60% of the participants, and 6 were impaired in over 75% (Fig. 2). The 6 most impaired functions were related to exercise tolerance functions (b455), urination functions (b620), muscle power functions (b730), motor reflex functions (b750), control of voluntary movement functions (b760) and gait pattern functions (b770). The most severe impairments were found in muscle power (b730) (2.6% were assessed as totally impaired, 7.9% severely impaired, 37.7% moderately impaired and 43.7% lightly impaired), control of voluntary movements functions (b760) (15.2% severely impaired, 32.5% moderately impaired and 41.1% lightly impaired) and gait pattern functions (b770) (1.3% were assessed as totally impaired, 11.3% severely impaired, 23.3% moderately impaired and 55.0% lightly impaired). The complete evaluation of b-categories is shown in Table SIII.

Six of 8 b-categories also included in the Brief ICF Core Set for MS were found to be impaired in at least 60% of the participants (Fig. 2). The remaining 2 functions of b-categories, b152 (emotional functions) and b164 (higher-level cognitive functions), were impaired in 46.4% and 35.1%, respectively (Table SIII).

**Fig. 3:**
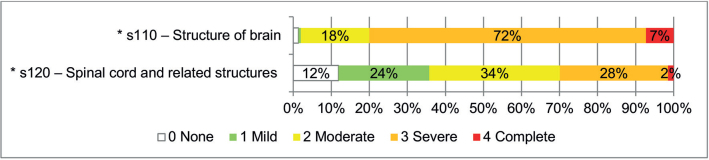
Body Structure impairments according to the Comprehensive International Classification of Functioning, Disability and Health (ICF) Core Set for multiple sclerosis (MS) (N_s110_ = 151 and N_s120_ = 67) are presented as percentages. *s-categories included in the Brief ICF Core Set for MS.

*Body Structures*. Brain structures (s110) were impaired in 148 participants (7.3% were totally impaired, 72.7% severely impaired, 17.9% moderately impaired and 0.7% lightly impaired), and spinal cord-related structures (s120) were impaired in 59 participants out of the 67 for whom this assessment was conducted (1.5% totally impaired, 28.4% severely impaired, 34.3% moderately impaired and 23.9% lightly impaired) (Fig. 3).

**Fig. 4:**
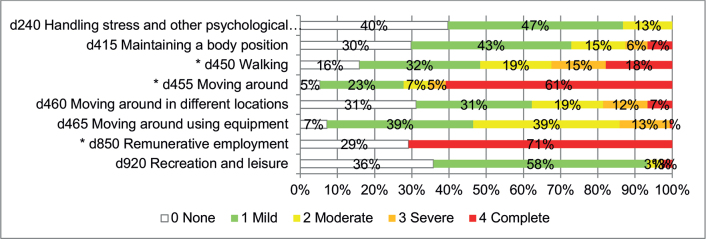
Impairments in Activities and Participation according to the Comprehensive International Classification of Functioning, Disability and Health (ICF) Core Set for multiple sclerosis (MS) (*N* = 151, except for d465 *N* = 97) in over 60% of the participants are presented as percentages. *d-categories included in the Brief ICF Core Set for MS.

*Activities and Participation*. Among 53 Activities and Participation categories, 8 were impaired in over 60% of the participants, and 3 were impaired in over 75% (Fig. 4). The latter categories were related to walking (d450) (17.9% totally impaired, 14.6% severely impaired, 19.2% moderately impaired and 32.5% lightly impaired), moving around (d455) (60.9% totally impaired, 4.6% severely impaired, 6.6% moderately impaired and 22.5% lightly impaired) or moving around using equipment (d465) (1.0% totally impaired, 13.4% severely impaired, 39.2% moderately impaired and 39.2% lightly impaired). The complete evaluation of d-categories is shown in Table SIII.

Three of the 6 d-categories included in the Brief ICF Core Set for MS were found to be impaired in at least 60% of the participants (Fig. 4). The remaining 3 d-categories d175 (solving problem), d230 (carrying out daily routine) and d760 (family relationships) were impaired in 14.6%, 12.6% and 27.8%, respectively (Table SIII).

**Fig. 5:**
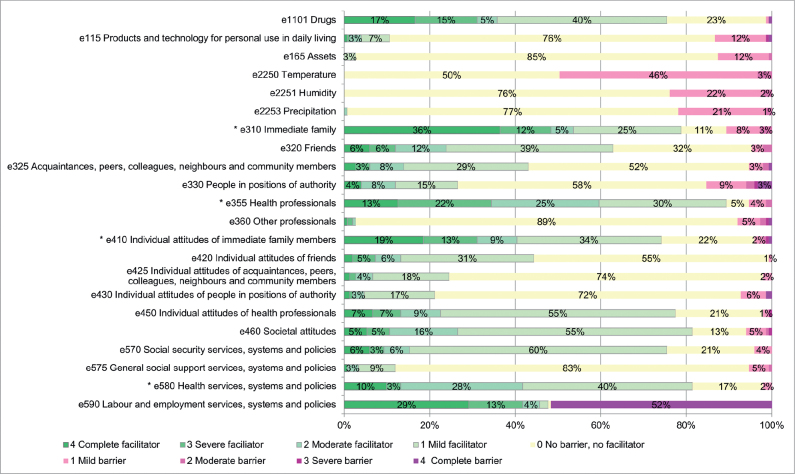
Environmental Factors and barriers/facilitators according to the Comprehensive International Classification of Functioning, Disability and Health (ICF) Core Set for multiple sclerosis (MS) are presented as percentages. *e-categories included in the Brief ICF Core Set for MS. Factors containing over 90% of the values either “no barrier or barrier is very low 0-4%” or “no or very low facilitator 0-4%” are excluded.

*Environmental Factors*. Thirty-seven Environmental Factors categories were evaluated, and those containing over 90% of the values either “no barrier or barrier is very low 0–4%” or “no or very low facilitator 0–4%” were excluded from Fig. 5. The results show that most of the e-categories were facilitators, especially drugs (e1101), immediate family (e310) and individual attitudes of immediate family members (e410), friends (e320), health professionals (e355) and individual attitudes of health professionals (e450), health services, systems and policies (e580). The following Environmental Factors were identified as barriers: temperature (e2250), humidity (e2251) and precipitation (e2253). Employment-associated factors were assessed as both facilitators and barriers (e590). The complete evaluation of e-categories is presented in Table SIII.

All 4 Environmental Factors categories included in the Brief ICF Core Set for MS were found to be facilitators (Fig. 5).

## DISCUSSION

The results of this descriptive cross-sectional single-centre study of 151 participants with MS show that a major part of the b- and d-categories assessed overlap with those selected for the Brief ICF Core Set for MS. This indicates the importance of these categories when assessing disability in MS. However, b164 (higher-level cognitive functions) was found to be impaired in only 35.1% of the participants. This corresponds with our previous report, in which 17% of the participants demonstrated impairment when using the Brief ICF Core Set for MS (15). Furthermore, b144 (memory functions) was impaired in 70% of participants, indicating that the latter b-category might be a better choice when assessing cognitive impairments using the ICF tool. Many muscle power functions not included in the Brief ICF Core Set for MS were found to be impaired in a large number (> 70%) of participants: exercise tolerance functions (b455), motor reflex functions (b750), control of voluntary movement functions (b760), moving around using equipment (d465), muscle endurance functions (b740) and maintaining a body position (d415).

Among specific mental functions related to the feeling and affective components of the processes of the mind, b126 (temperament and personality factors) evaluates the general mental functions of the constitutional disposition of the individual to react in a particular way to situations, including the set of mental characteristics that makes the individual distinct from others, while b152 (emotional functions) evaluates functions related to anxiety and depression levels (Appendix S1). Concluding from these results we suggest the replacement of b164 (higher-level cognitive functions) with b144 (memory functions) and b152 (emotional functions) with b126 (temperament and personality factors).

Among d-categories, the most impairments were found in activities associated with moving (d455, d460, d465), walking (d450), maintaining body position (d415) and remunerative employment (d850). These activities were clearly impaired and are present in both the Comprehensive ICF Core Set for MS and the Brief ICF Core Set for MS. The remaining 3 activities in the Brief ICF Core Set for MS, such as solving problems (d175), carrying out daily routines (d230) and family relationships (d760), were impaired in fewer participants. Furthermore, a large number of participants were assessed to have impairments in d240 (handling stress and other psychological demands) and d920 (recreation and leisure), which are not included in the Brief ICF Core Set for MS. Taken together, the results suggest that these 2 activities should be considered for inclusion in the Brief ICF Core Set for MS.

All 4 e-categories in the Brief ICF Core Set were facilitators (e310, e355, e410 and e580). However, the results indicate that e2250 (temperature) is a clear barrier and that e590 (labour and employment systems) is both a facilitator and a barrier. Forty-seven percent of participants were employed, and 85% had a tertiary education. For middle-aged participants still active in the labour market, e590 (labour and employment systems) is an important e-category and should be included in the Brief ICF Core Set for MS.

In MS research and clinical practice, the ICF is accepted, but not yet widely used. The ICF has been used for MS symptom evaluation (17), for assessment of activity and participation using the patients’ perspective (10), for functioning and disability rating (18) and for evaluation of physical therapy outcome (19). In a recently published study of patients with MS from DWACO, we found that b164 (higher-level cognitive functions) was predictive of MS progression within 1 year (15). Validation studies on the Comprehensive ICF Core Set for MS as a tool from a physician perspective remain limited. To our knowledge, this study is the first to present impairments according to the Comprehensive ICF Core Set for MS from the physician perspective in a working capacity assessment setting.

Taken together, the results of the current study show additional ICF categories that were frequently impaired in the current study cohort, which might be important and relevant for the Brief ICF Core Set (Table II). The process, however, needs to be validated in other cohorts and settings in order to be internationally accepted. On the other hand, these categories might also be important when evaluating work ability, which needs to be further analysed in future studies.

**Table II:** Suggested International Classification of Functioning, Disability and Health (ICF) categories for the Brief ICF Core Set for multiple sclerosis

ICF categories	Impairments or barriers (%)
b126 Temperament and personality functions	74.8
b144 Memory functions	70.2
b455 Exercise tolerance functions	94.0
b5500 Body temperature	68.2
b740 Muscle endurance functions	73.5
b750 Motor reflex functions	81.5
b760 Control of voluntary movement functions	88.7
d415 Maintaining a body position	70.2
d465 Moving around using equipment	92.8
e2250 Temperature	49.7
e590 Labour and employment services, systems and policies	51.7

### Study limitations

We acknowledge the selection bias in this study, since only participants referred for work capacity assessment at DWCAO under the Ministry of Social Security and Labour of the Republic of Lithuania were recruited. The evaluation of ICF categories was performed by the same evaluator, which might be considered both a strength and a limitation. However, assessments of Body Functions, Activity and Participation, and Environmental Factors were performed using a strict study protocol. Another clear limitation was the use of telephone interviews instead of physical appointments due to COVID-19 pandemic restrictions. However, telemedicine is becoming more popular in clinical practice, and the results of this study indicate that data collection can be conducted through this method despite its difference from classical appointments.

### Study strengths

ICF evaluations were applied to cover the multidimensional aspects of MS and were based on both the strict protocol for the evaluation of each category and journal recordings of medical history available from DWACO.

### Conclusion

The results of this study reveal that several ICF categories should be considered for inclusion in the Brief ICF Core Set for MS for the further development of ICF applications in clinical settings from a clinician’s perspective.

## Supplementary Material

REVISION OF THE BRIEF INTERNATIONAL CLASSIFICATION OF FUNCTIONING, DISABILITY AND HEALTH CORE SET FOR MULTIPLE SCLEROSIS: A STUDY OF THE COMPREHENSIVE ICF CORE SET FOR MULTIPLE SCLEROSIS WITH PARTICIPANTS REFERRED FOR WORK ABILITY ASSESSMENT

REVISION OF THE BRIEF INTERNATIONAL CLASSIFICATION OF FUNCTIONING, DISABILITY AND HEALTH CORE SET FOR MULTIPLE SCLEROSIS: A STUDY OF THE COMPREHENSIVE ICF CORE SET FOR MULTIPLE SCLEROSIS WITH PARTICIPANTS REFERRED FOR WORK ABILITY ASSESSMENT
