# Supplementary material for: REVISION OF THE BRIEF INTERNATIONAL CLASSIFICATION OF FUNCTIONING, DISABILITY AND HEALTH CORE SET FOR MULTIPLE SCLEROSIS: A STUDY OF THE COMPREHENSIVE ICF CORE SET FOR MULTIPLE SCLEROSIS WITH PARTICIPANTS REFERRED FOR WORK ABILITY ASSESSMENT
Source: J Rehabil Med. 2024 Mar 7;56:19671. doi: 10.2340/jrm.v56.19671 (PMC10938140; doi:10.2340/jrm.v56.19671)
Supplement: REVISION OF THE BRIEF INTERNATIONAL CLASSIFICATION OF FUNCTIONING, DISABILITY AND HEALTH CORE SET FOR MULTIPLE SCLEROSIS: A STUDY OF THE COMPREHENSIVE ICF CORE SET FOR MULTIPLE SCLEROSIS WITH PARTICIPANTS REFERRED FOR WORK ABILITY ASSESSMENT [file JRM-56-19671-s1.pdf]

Supplementary material has been published as submitted. It has not been copyedited, typeset or checked for scientific content by Journal of Rehabilitation Medicine

Appendix S1

A detailed description of the Comprehensive ICF core set for MS category assessment

BODY FUNCTIONS

b114 Orientation functions

General mental functions of knowing and ascertaining one's relation to self, to others, to time and to one's surroundings.

Inclusions: Functions of orientation to time, place and person; orientation to self and others; disorientation to time, place and person

b1140 Orientation to time

Mental functions that produce awareness of day, date, month and year.

b1141 Orientation to place

Mental functions that produce awareness of one's location, such as one's immediate surroundings, one's town or country.

b1142 Orientation to person

Mental functions that produce awareness of one's own identity and of individuals in the immediate environment.

0– No impairment. The person has good orientation to time, place or person.

1- Mild impairment. The person´s orientation *to time, place or person* is mildly impaired but self-adapting to the environment.

2- The person´s orientation *to time, place or person* is moderately impaired. There is unstable orientation and requirements of aids (e.g., maps and notes) and episodic help from another person.

3- The person's orientation is severely impaired, with persistent difficulty in orienting oneself to time or place, or orientation is impaired towards oneself and others. There is a constant need for help from another person.

4- Complete impairment. The person cannot be oriented to time, place, self and others. There is a requirement for ongoing care.

b126 Temperament and personality functions

General mental functions of the constitutional disposition of the individual to react in a particular way to situations, including the set of mental characteristics that makes the individual distinct from others.

Inclusions: Functions of extraversion, introversion, agreeableness, conscientiousness, psychic and emotional stability, openness to experience, optimism, novelty seeking, confidence, trustworthiness

b1263 Psychic stability

Mental functions that produce a personal disposition that is even-tempered, calm and composed, in contrast to being irritable, worried, erratic and moody.

0- No impairment. The person is even-tempered, calm and composed and can adapt successfully in social, work and family environments.

1- Mild impairment. The person is occasionally irritable, worried, erratic and moody, but the person adapts to the environment.

2- Moderate impairment. The person is occasionally irritable, worried, erratic and moody, with complicated adaptation to the environment.

3- Severe impairment. The person´s personality traits (irritable, worried, erratic and moody) complicate adaptation in such a way that the person finds it very difficult to react to the environment and needs constant help from another person.

4- Complete impairment. The person's personality features interfere with adaptation, and clinical personality disorders are observed.

b1264 Openness to experience

Mental functions that produce a personal disposition that is curious, imaginative, inquisitive and experience-seeking, in contrast to being stagnant, inattentive and emotionally inexpressive.

0- No impairment. The person is curious, imaginative, inquisitive and able to adapt successfully in social, work and family environments.

1- Mild impairment. The person is occasionally stagnant, inattentive and emotionally inexpressive, but the person adapts to the environment. There are traits for adaptation. Some personal characteristics are difficult, but the person adapts to the environment.

2- Moderate impairment. The person is occasionally stagnant, inattentive and emotionally inexpressive, and finds it difficult to adapt to the environment.

3- Severe impairment. The person´s personality traits (stagnant, inattentive and emotionally inexpressive) complicate adaptation in such a way that the person finds it very difficult to react to the environment and needs constant help from another person.

4- Complete impairment. The person's personality features interfere with adaptation, and clinical personality disorders are observed.

b1266 Confidence

Mental functions that produce a personal disposition that is self-assured, bold and assertive, in contrast to being timid, insecure and self-effacing.

0- No impairment. The person is self-assured, bold and assertive and able to adapt successfully in social, work and family environments.
1- Mild impairment. The person is timid, insecure and self-effacing, but adapts to the environment.
2- Moderate impairment. The person is timid, insecure and self-effacing, and these traits complicate adaptation to the environment.
3- Severe impairment. The person´s personality traits (timid, insecure and self-effacing) complicate adaptation in such a way that the person finds it very difficult to adapt to the environment and needs constant help from another person.
4- Complete impairment. The person's personality features interfere with adaptation, and clinical personality disorders are observed.

b 130 Energy and drive functions

General mental functions of physiological and psychological mechanisms that cause the individual to move towards satisfying specific needs and general goals in a persistent manner.

Inclusions: Functions of energy level, motivation, appetite, craving (including craving for substances that can be abused), and impulse control.

b 1300 Energy level

Mental functions that produce vigour and stamina.

0- No impairment. The person has their own goals and strategies to achieve them and can consider the arguments, make a decision and persist and execute the goals independently.
1- Mild impairment. The person wants to, can, and has strategies to perform a voluntary action to achieve their goal and can consider the arguments but has difficulties making a decision, and achieving a goal takes longer.
2- Moderate impairment. The person's goals are poor, but there is a capacity to achieve them, and there is a need for assistance from other persons to understand the purpose and to argue before making a decision, which takes longer.
3- Severe impairment. The person lacks initiatives and goals or is not able to realize them without help, i.e., to present goals and supervise an execution (for example, to perform work under supervision).
4- Complete impairment. The person is passive, lacks goals, is inactive, spends most of their time lying in bed, and their interests are limited to maintaining physiological functions. Overall indifference.

b1301 Motivation

Mental functions that produce the incentive to act. The conscious or unconscious driving force for action.

0- No impairment. The person´s drive functions are intact, and the person is motivated and achieves their own goals.

1- Mild impairment. The person´s motivation action is slightly impaired, the person is motivated, strives to achieve their own goals, but does not always achieve the result and/or does not reach a certain level of satisfaction.
2- Moderate impairment. The person´s motivation is moderately impaired, and they are motivated to act but do not have the ability and require episodic help.
3- In severe impairment. The person has high impairment of the function that motivates action. The person is not motivated to act, cannot meet their needs, and requires ongoing support.
4- Complete impairment. The person has total impairments of the function that motivates action, or the person is unmotivated.

b134 Sleep functions

General mental functions of periodic, reversible and selective physical and mental disengagement from one's immediate environment accompanied by characteristic physiological changes.

Inclusions: functions of the amount of sleep and onset, maintenance and quality of sleep, functions involving the sleep cycle, such as in insomnia, hypersomnia and narcolepsy

b1340 Amount of sleep

Mental functions involved in the time spent in the state of sleep in the diurnal cycle or circadian rhythm.

0- No impairment. The person falls asleep without medication, sleeps through the night and wakes up rested in the morning.
1- Mild impairment. The person takes light medication (sedatives, herbal teas), which allows them to sleep through the night and wake up rested in the morning.
2- Moderate impairment. The person sleeps up to 7 hours, has erratic/occasional use of sleeping pills to fall asleep and stay asleep throughout the night, and wakes up rested in the morning.
3- Severe impairment. The person sleeps up to 6 hours, takes sleeping pills, and does not feel rested in the morning.
4- Complete impairment. The person sleeps up to 5 hours and takes strong sleep medication but still has difficulty falling asleep, wakes up during the night, and feels restless in the morning.

b1341 Onset of sleep

Mental functions that produce the transition between wakefulness and sleep.

0- No impairment. The person falls asleep without medication and sleeps through the night.
1- Mild impairment. The person takes light medication (sedatives, herbal teas), which allows them to sleep through the night.
2- Moderate impairment. The person episodically uses sleeping pills to fall asleep.
3- Severe impairment. The person constantly uses sleeping pills to fall asleep.

4- Complete impairment. The person takes strong medication but still has difficulty falling asleep and wakes up during the night.

b140 Attention functions

Specific mental functions of focusing on an external stimulus or internal experience for the required period.

Inclusions: Functions of sustaining attention, shifting attention, dividing attention, sharing attention, concentration, and distractibility.

b1400 Sustaining attention

Mental functions that produce concentration for the period needed.

0- No impairment. The person sustains attention and focuses their mental activity on a specific object to the exclusion of everything else.
1- Mild impairment. The person has mild impairment in sustaining attention and can concentrate on external stimuli by using personal experience only for the required time.
2- Moderate impairment. The person has moderate impairment in sustaining attention. The person can focus for a short time (approximately 30 min) and concentrate on external stimuli by using personal experience. The person is quickly distracted by weak stimuli.
3- Severe impairment. The person has severe impairment in sustaining attention. The person cannot maintain focus for longer than 10 min, has difficulties speaking and thinking, makes pauses and mistakes, is easily distracted by new stimuli, and needs constant help from another person.
4- Complete impairment. The person has a complete deficit in sustaining attention and cannot pay attention.

b1401 Shifting attention

Mental functions that permit refocusing concentration from one stimulus to another.

0- No impairment. The person can shift attention and refocus mental activity on a specific object to the exclusion of everything else.
1- Mild impairment. The person has mild shifting attentional impairment, can refocus concentration from one stimulus to another, attention is distracted only by strong stimuli.
2- Moderate impairment: the person has moderate impairment in shifting attention and can refocus concentration from one stimulus to another, but attention is quickly distracted by weak stimuli.
3- Severe impairment: the person has severe impairment in shifting attention and cannot refocus concentration from one stimulus to another. The person has difficulties speaking and thinking, makes

pauses and mistakes, is easily distracted by new stimuli, cannot switch from one thought or activity to another, and needs help from another person.

4- Complete impairment. The person has a complete deficit in shifting attention. The person cannot pay attention to questions.

b144 Memory functions

Specific mental functions of registering and storing information and retrieving it as needed.

Inclusions: Functions of short-term and long-term memory, immediate, recent and remote memory, memory span, retrieval of memory, remembering; functions used in recalling and learning, such as in nominal, selective and dissociative amnesia.

b1440 Short-term memory

Mental functions that produce a temporary, disruptable memory store of approximately 30 seconds in duration from which information is lost if not consolidated into long-term memory.

0- No impairment. The person does not have short-term memory impairments.

1- Mild short-term memory impairment. The person has good memorization only when interested, retains important information about a favourite subject or occupation, and retrieves information only when trying to remember something or involuntarily and spontaneously.

2- Moderate short-term memory impairment. The person has a weakened ability to recall and remember information and needs aids (i.e., notes).

3- Severe short-term memory impairment. The person has progressive memory impairments, is diagnosed with amnesia, and requires aids (notes) and help from another person.

4- Complete short-term memory impairment. The person is helpless due to severe short-term memory and recall impairment.

b1441 Long-term memory

Mental functions that produce a memory system permitting the long-term storage of information from short-term memory and both autobiographical memory of past events and semantic memory of language and facts.

0- No impairment. The person does not have memory impairments.

1- Mild long-term memory impairment. The person has good memorization only when interested, retains important information about a favourite subject or occupation, and retrieves only when trying to remember something or involuntarily and spontaneously.

2- Moderate long-term memory impairment. The person has a weakened ability to memorize and recall information and needs aids (i.e., notes, photographs).

3- Severe long-term memory impairment. The person has progressive memory impairments, is diagnosed with amnesia, and requires aids (i.e., notes) and help from another person.

4- Complete long-term memory impairment. The person is helpless due to severe long-term memory and recall impairment.

b152 Emotional functions (G)

Specific mental functions related to the feeling and affective components of the processes of the mind.

Inclusions: Functions of the appropriateness of emotion, regulation and range of emotion, affect, sadness, happiness, love, fear, anger, hate, tension, anxiety, joy and sorrow, lability of emotion, and flattening of affect.

b1520 Appropriateness of emotion

Mental functions that produce congruence of feeling or affect with the situation, such as happiness when receiving good news.

0- No impairment. The person does not have any emotional impairments, and emotional expressions are adequate.
1- Mild impairment. The person has adequate emotions but increased sensitivity to a fact, news, or event. The person can maintain a stable mood.
2- Moderate impairment. The person has increased emotional reactions to a fact, news, or event with emotional variation. Episodically, the person needs support when assessing a situation. The person can regulate their own emotions and feelings after a proper assessment of the situation.
3- Severe impairment. The person has poor emotional variation, inadequate or opposite reactions to a fact, news, or event, and there is a need for continuous support.
4- Complete impairment. The person has poor and inadvertent emotions. There is a lack of feelings and impaired emotional regulation and expression.

b156 Perceptual functions

Specific mental functions of recognizing and interpreting sensory stimuli.

Inclusions: Functions of auditory, visual, olfactory, gustatory, tactile and visuospatial perception, such as a hallucination or illusion

b1560 Auditory perception

Mental functions involved in discriminating sounds, tones, pitches and other acoustic stimuli.

0- No impairment. The person does not have auditory perception impairments.
1- Mild auditory perception impairment. The person does not experience unpleasant sensations.
2- The person has moderate auditory perception impairment, causing very unpleasant sensations and experiences.
3- Severe auditory perception impairment. The person does not perceive audible sounds and can cognize and perceive auditory phenomena with the help of another person.

4- Complete auditory perception impairment. The person cannot perceive sounds and is completely dependent on another person.

b164 Higher-level cognitive functions.

Specific mental functions especially dependent on the frontal lobes of the brain, including complex goal-directed behaviours such as decision-making, abstract thinking, planning and conducting plans, mental flexibility, and deciding which behaviours are appropriate under what circumstances, often called executive functions.

Inclusions: Functions of abstraction and organization of ideas, time management, insight and judgement, concept formation, categorization and cognitive flexibility.

b1641 Organization and planning

Mental functions of coordinating parts into a whole, of systematizing; the mental function involved in developing a method of proceeding or acting.

0- The person does not have executive impairments or difficulties.
1- The person has slight organization and planning impairment but with no interference with purposeful activities.
2- The person has moderate organization and planning impairment. The person can perform purposeful activities with aids and/or support from another person.
3- The person has severe organization and planning impairment. There is a lack of independent executive functions, and there is a constant need for aids and/or support from another person.
4- The person has total impairment in executive functions.

b1642 Time management

Mental functions of ordering events in chronological sequence, allocating amounts of time to events and activities.

0- The person does not have executive impairments or difficulties.
1- The person has slight time management impairment but without interference with purposeful activities.
2- The person has moderate time management impairment. The person can perform purposeful activities with aids and/or support from another person.
3- The person has severe time management impairment. There is a lack of independent executive functions, and there is an overall need for aids and/or support from another person.
4- The person has total impairment in executive time management functions.

b1646 Problem-solving

Mental functions of identifying, analysing and integrating incongruent or conflicting information into a solution (e.g., after traffic accident).

0- The person does not have executive impairments or difficulties.
1- The person has slight problem-solving impairment, but without interruption of purposeful activities.
2- The person has moderate problem-solving impairment. The person can perform purposeful activities with aids and/or support from another person.
3- The person has severe problem-solving impairment. There is a lack of independent executive functions and a constant need for aids and/or support from another person.
4- The person has total impairments in executive functions.

b210 Seeing functions.

Sensory functions related to sensing the presence of light and sensing the form, size, shape, and colour of visual stimuli.

Inclusions: visual acuity functions, visual field functions, quality of vision, functions of sensing light and colour, visual acuity of distant and near vision, monocular and binocular vision, visual picture quality, impairments such as myopia, hypermetropia, astigmatism, hemianopia, colour blindness, tunnel vision, central and peripheral scotoma, diplopia, night blindness, and impaired adaptability to light.

b2100 Visual activity functions

Seeing functions of sensing form and contour, both binocular and monocular, for both distant and near vision.

0- No impairment. The person can independently and safely perform activities without an aid (spectacles) or another person´s support and without vision difficulties.
1- Mild visual activity function impairment. The person performs visual activities independently and safely with the occasional need for aids.
2- Moderate visual activity function impairment. The person performs visual activities independently and safely with permanent need of aids and/or sometimes another person´s support.
3- Severe visual activity function impairment. The person performs visual activities with great difficulties and with a permanent need for aids and support from another person.
4- Complete impairment of visual activity functions. Total blindness, completely dependent on other persons.

b260 Proprioceptive function

Sensory functions of sensing the relative position of body parts.

Inclusions: Functions of statesthesia and kinaesthesia.

0- No impairment. The person does not have any impairments in proprioception.

1- Mild impairment. The person has inaccurate sensation of his or her own finger position and movement.

2- Moderate impairment. The person has no sense of his or her own finger position and movement.

3- Severe impairment. The person senses only his or her own limb position and movement.

4- Complete impairment. The person cannot identify the position of their own limbs.

b265 Touch function

Sensory functions of sensing surfaces and their texture or quality.

Inclusions: Functions of touching, feeling of touch. impairments such as numbness, anaesthesia, tingling, paraesthesia and hyperaesthesia.

0- No impairment. The person does not have disturbances in feeling the touch.

1- Mild impairment. The person has an inaccurate sensation of tactile touch.

2- Moderate impairment. The person has decreased feeling of touch (hypoesthesia).

3- Severe impairment. The person has sensation only after strong touch stimuli.

4- Complete impairment. The person does not feel touch (anaesthesia to touch).

b270 Sensory functions related to temperature and other stimuli

Sensory functions of sensing temperature, vibration, pressure and noxious stimulus.

Inclusions: Functions of sensitivity to temperature, vibration, shaking or oscillation, superficial pressure, deep pressure, burning sensation or a noxious stimulus.

b2700 Sensitivity to temperature

Sensory functions of sensing cold and heat.

0- No impairment. The person does not have impairments in feeling temperature.

1- Mild impairment. The person has inaccurate sensation to temperature.

2- Moderate impairment. The person has decreased feelings of temperature.

3- Severe impairment. The person has a sensation of temperature only with strong stimuli.

4- Complete impairment. The person does not feel temperature (anaesthesia to temperature).

b2701 Sensitivity to vibration

Sensory functions of sensing shaking or oscillation (e.g., sensing if the mixer is on)

0- No impairment. The person does not have impairments in feeling vibration.

1- Mild impairment. The person has inaccurate sensation to vibration.

2- Moderate impairment. The person has decreased feelings of vibration.
3- Severe impairment. The person has a sensation of temperature only with strong stimuli.
4- Complete impairment. The person does not feel vibration (anaesthesia to vibration stimuli).

b280 Sensation of pain

Sensation of unpleasant feelings indicating potential or actual damage to some body structure.

Inclusions: sensations of generalized or localized pain in one or more body parts, pain in a dermatome, stabbing pain, burning pain, dull pain, and aching pain, impairments such as myalgia, analgesia and hyperalgesia.

b2800 Generalized pain

Sensation of unpleasant feelings indicating potential or actual damage to some body structure felt all over or throughout the body.

0- No impairment. The person does not feel pain in the body.
1- Mild impairment. The person infrequently feels slight pain throughout the body or pain only occurs during movement.
2- Moderate impairment. The person occasionally feels pain throughout the body, with brief pain interference during activity or movement.
3- Severe impairment. The person constantly feels pain throughout the body, which stops the activity. There is a need for pain medication.
4- Complete impairment. The person experiences severe pain throughout the body that prevents the performance of any activity.

b2801 Pain in body part

Sensation of unpleasant feelings indicating potential or actual damage to some body structure felt in a specific part or parts of the body.

0- No impairment. The person does not feel pain in the body.
1- Mild impairment. The person feels mild pain in one part of the body or pain only occurs during movement.
2- Moderate impairment. The person feels pain in one part of the body, with brief pain interference during activity or movement.
3- Severe impairment. The person feels pain in one part of the body, which stops the activity. There is a need for pain medication.
4- Complete impairment. The person experiences very severe pain in one part of the body that prevents the performance of any activity.

b310 Voice functions

Functions of the production of various sounds by the passage of air through the larynx.

Inclusions: Functions of production and quality of voice, functions of phonation, pitch, loudness and other qualities of voice, impairments such as aphonia, dysphonia, hoarseness, hypernasality and hyponasality.

b3100 Production of voice

Functions of the production of sound made through coordination of the larynx and surrounding muscles with the respiratory system.

Inclusions: Functions of phonation, loudness, and impairment of aphonia.

0- The person does not have any voice impairments.
1- The person has mild impairments. Voice quality or voice production might be slightly impaired.
2- Moderate impairment. The person has impaired voice production. The voice is weak and articulation is impaired (i.e., the person speaks with low volume and the voice is monotone).
3- Severe impairment. The person has impaired voice quality, and only isolated noises are produced.
4- The person has completely impaired voice function and cannot vocalize.

b320 Articulation functions

Functions of speech sound production.

Inclusions: Functions of enunciation, articulation of phonemes. spastic, ataxic, flaccid dysarthria, anarthria

0- The person normally articulates and produces speech freely.
1- The person has mild impairments. There is slight impairment in pronunciation. The person's speech is understandable, and the speech impairments are almost imperceptible.
2- The person has moderately impaired pronunciation and blurred dysarthria, and their speech is hard for others to understand.
3- The person has severe impairments in pronunciation, and dysarthria, making speech barely understandable to others.
4- The person has completely impaired pronunciation, and there is no speech or anarthria.

b330 Fluency and rhythm of speech functions

Functions of the production of flow and tempo of speech.

Inclusions: Functions of fluency, rhythm, speed and melody of speech, prosody and intonation, impairments such as stuttering, stammering, cluttering, bradylalia and tachylalia.

b3300 Fluency of speech

Functions of the production of smooth, uninterrupted flow of speech.

Inclusions: Functions of smooth connection of speech. Impairments such as stuttering, stammering, cluttering, dysfluency, repetition of sounds, words or parts of words and irregular breaks in speech.

0- The person speaks fluently without interruptions.
1- The person has mild impairments in speech fluency and rhythm. The person finds the right words to express his or her own thoughts; however, he or she may talk in excitement or in a hurry.

2- The person has moderate impairment in speech fluency and rhythm. There is episodic difficulty in selecting the right words and forming grammatically correct sentences. However, the speech is understandable to others.
3- The person has severe impairment in speech fluency and rhythm with an impaired ability to select appropriate words and grammatically correct sentences. The person has an impaired ability to react adequately and to respond to questions.
4- The person has completely impaired speech and does not speak.

b3302 Speed of speech

Functions of the rate of speech production.

Inclusions: Impairments such as bradylalia and tachylalia.

0- The person speaks at a normal speed.
1- The person has mild speed of speech impairments, the pace is not always natural, but the speech is fluent and coherent.
2- The person has moderate speed of speech impairment, unnatural pace, speaking slowly or fast, but coherently.
3- The person has severe speed of speech impairments, such as bradylalia or tachylalia.
4- The person has completely impaired speech. Conversation is not supported due to very slow or very fast speech rates.

b455 Exercise tolerance functions

Functions related to respiratory and cardiovascular capacity as required for enduring physical exertion.

Inclusions: Functions of physical endurance, aerobic capacity, stamina and fatigability.

b4550 General physical endurance

Functions related to the general level of tolerance of physical exercise or stamina (e.g., to get to the 2nd floor).

0- No impairment. The person has good physical endurance.
1- Mild general physical endurance impairment. The person gets tired after 30 min of intense activity.
2- Moderate general physical endurance impairment. The person gets tired after self-care and other vigorous activities of 15 min.
3- Severe general physical endurance impairment. The person becomes tired while performing routine self-care activities (dressing, showering, eating).
4- With complete impairment. The person has interruptions in daily self-care, sometimes even during one activity (e.g., eating).

b4552 Fatigability

Functions related to susceptibility to fatigue at any level of exertion (e.g., staying at home and not going for walks with your family).

0- No impairment. The person is not affected by fatigue.
1- Mild impairment. The person complains of irregular fatigue when asked.
2- Moderate impairment. The person complains of episodic fatigue to medical staff or other persons.
3- Severe impairment. The person complains of constant fatigue to medical staff and takes medication (for example amantadine).
4- Complete impairment. The person constantly feels tired despite taking medication.

b5104 Salivation

Function of saliva production within the mouth.

0- No impairment. The person produces a normal amount of saliva.
1- Mild impairment. The person has a wet pillow at night or an occasional dry mouth.
2- Moderate impairment. The person is bothered by salivation during the day.
3- Severe impairment. The person uses prescribed medication for salivation.
4- Complete impairment. The person has complete disturbance of salivation.

b5105 Swallowing

Functions of clearing the food and drink through the oral cavity, pharynx and oesophagus into the stomach at an appropriate rate and speed.

Inclusions: Oral, pharyngeal or oesophageal dysphagia, impairments in oesophageal passage of food.

0- No impairment. The person does not have any disturbed swallowing.
1- Mild impairment. The person has slight difficulties in swallowing food.
2- Moderate impairment. The person has difficulty swallowing liquid food.
3- Severe impairment. The person is able to eat solid food only.
4- Complete impairment. The person cannot eat without aids and has a nasogastric tube.

b525 Defecation functions

Functions of elimination of wastes and undigested food as faeces and related functions.

Inclusions: Functions of elimination, faecal consistency, frequency of defecation, faecal continence, flatulence, impairments such as constipation, diarrhoea, watery stool and anal sphincter incompetence or incontinence.

b5250 Elimination of faeces

Functions of the elimination of waste from the rectum, including the functions of contraction of the abdominal muscles.

0- No impairment. The person does not have any problems with faecal elimination.

1- Mild impairment. The person has slowed faecal elimination, and obstipation is present.
2- Moderate impairment. The person has slowed faecal elimination, frequent obstipation, and medication is needed.
3- Severe impairment. The person has involuntary stool elimination, but there is a sense of urge.
4- Complete impairment. The person has involuntary elimination of faeces and/or does not feel the urge, and there is a need for constant enemas.

b5253 Faecal continence

Functions involved in voluntary control over the elimination function.

0- No impairment. The person has control of defecation.
1- Mild faecal continence impairment. The person has good bowel control but occasional incontinence (no more than once every 10 days).
2- Moderate faecal continence impairment. The person voluntarily controls bowel movements but has frequent incontinence (at least once a week).
3- Severe faecal continence impairment. The person defecates involuntarily but feels the urge.
4- Complete impairment, involuntary defecation, and constant use of nappies.

b5500 Body temperature

Functions involved in regulating the core temperature of the body.

Inclusion: Impairments such as hyperthermia or hypothermia.

b5501 Maintenance of body temperature

Functions involved in maintaining optimal body temperature as environmental temperature changes.

Inclusion: Tolerance to heat or cold.

0- No impairment. The person has no impairment of body temperature maintenance.
1- Mild impairment. The person has slight disturbance in body temperature maintenance, only noticeable with changes in ambient temperature.
2- Moderate impairment. The person has moderate impairment of body temperature maintenance, worsening of symptoms in the heat, and reduced endurance to cold and/or heat.
3- Severe impairment. The person has severe impairment in maintaining body temperature, is unable to tolerate heat, has worsening symptoms, and has no tolerance of cold and/or heat.
4- Complete impairment in maintaining body temperature.

b620 Urination functions

Functions of discharge of urine from the urinary bladder.

Inclusions: Functions of urination, frequency of urination, and urinary continence, impairments such as stress, urge, reflex, overflow, continuous incontinence, dribbling, automatic bladder, polyuria, urinary retention, and urinary urgency.

b6200 Urination

Functions of voiding the urinary bladder.

Inclusions: Impairments such as in urine retention.

0- No impairment. The person has normal urination.
1- Mild impairment, urinary retention is seldom (no more than once per week) or the entire bladder is not emptied.
2- Moderate impairment. The person has episodic urinary retention.
3- Severe impairment. The person has frequent urinary retention.
4- Complete impairment. The person has constant urinary retention and needs catheterization.

b6202 Urinary continence

Functions of control over urination.

Inclusions: Impairments such as in stress, urge, reflex, continuous and mixed incontinence.

0- No impairment. The person does not have any urinary incontinence.
1- Mild impairment. The person has infrequent urinary incontinence or urgency, which occurs in stressful situations.
2- Moderate impairment. The person has mixed urinary incontinence and urgency.
3- Severe impairment. The person has urinary urgency and incontinence, resulting in urges and dribbling.
4- Complete impairment. The person has involuntarily urination and continuous incontinence and dribbling.

b730 Muscle power functions.

Functions related to the force generated by the contraction of a muscle or muscle groups. *Inclusions: Functions associated with the power of specific muscles and muscle groups, muscles of one limb, one side of the body, the lower half of the body, all limbs, the trunk, and the body as a whole. Impairments such as weakness of small muscles in the feet and hands, muscle paresis, muscle paralysis, monoplegia, hemiplegia, paraplegia, quadriplegia, and akinetic mutism. Functions related to the force generated by the contraction of a muscle or muscle groups.*

0- No impairment. The muscle or muscle group contraction power is 5 points according to Lovett's scale – normal muscle force.
1- Mild impairment. The muscle force is 4 points according to Lovett's scale – decreased power, there is resistance to gravity force and traction.

2- Moderate impairment. The muscle force is 3 points according to Lovett's scale – can overcome resistance to gravity force, but not traction.
3- Severe impairment. The muscle force is 1–2 points according to Lovett's scale – there is no resistance to gravity force, able to perform minimal movements.
4- Complete impairment. The person has plegia (0 points according to Lovett´s scale).

b735 Muscle tone functions

Functions related to the tension present in the resting muscles and the resistance offered when trying to move the muscles passively.

Inclusions: Functions associated with the tension of isolated muscles and muscle groups, muscles of one limb, one side of the body and the lower half of the body, muscles of all limbs, muscles of the trunk, and all muscles of the body. Impairments such as hypotonia, hypertonia and muscle spasticity.

0- No impairment, normal muscle tone.
1- Mild impairment. The person has a slight increase in muscle tone manifesting as weak retention or minimal resistance at the end of the range of movement, or an increase in muscle tone manifesting as a "curved knife" symptom, accompanied by minimal resistance in less than half of the movement (modified Ashworth scale score 1/1+).
2- Moderate impairment. The person has a moderate increase in muscle tone, with a more marked increase in muscle tone during most movements, but easy movement of the affected limb (modified Ashworth scale score 2); spasticity-reducing medication is used in the event of worsening.
3- Severe impairment. The person has a significant increase in muscle tone throughout the full range of movement, difficulty with passive movement (modified Ashworth scale score 3), constant use of spasticity-reducing medication.
4- Complete impairment. The affected limbs are rigid during extension and flexion movements (modified Ashworth score 4), and the affected limb is immobile during flexion or extension.

b740 Muscle endurance functions

Functions related to sustaining muscle contraction for the required period.

Inclusions: Functions associated with sustaining muscle contraction for isolated muscles and muscle groups and all muscles of the body; impairments such as myasthenia gravis.

0 - No impairment. The person has no disorder.
1 - Mild impairment. The person has muscle fatigue (in paresis).

2 - Moderate impairment. Muscle fatigue is observed after several exercises (with paresis, a positive Bare symptom in one limb).
3 - Severe impairment. Muscle fatigue is observed after minimal exercises (in deep paresis, a positive Bare symptom in two or more limbs).
4 - Complete impairment. No muscular endurance (in case of plegia).

b750 Motor reflex functions

Functions of involuntary contraction of muscles automatically induced by specific stimuli.

Inclusions: Functions of stretch motor reflex, automatic local joint reflex, reflexes generated by noxious stimuli and other exteroceptive stimuli, withdrawal reflex, biceps reflex, radius reflex, quadriceps reflex, patellar reflex, and ankle reflex.

0 - No impairment. All reflexes are present.
1 - Mild impairment. One of the reflexes is raised or lower.
2 - Moderate impairment. Two or three reflexes are present, and a positive Babinski reflex is present on one side.
3 - Severe impairment. All reflexes are raised/alive or low and positive Babinski reflex on both sides.
4 - Complete impairment. Reflexes not elicited.

b760 Control of voluntary movement functions

Functions associated with control over and coordination of voluntary movements.

Inclusions: Functions of control of simple voluntary movements and complex voluntary movements, coordination of voluntary movements, supportive functions of arm or leg, right–left motor coordination, eye–hand coordination, eye–foot coordination. Impairments such as control and coordination problems, e.g., dysdiadochokinesia.

b7602 Coordination of voluntary movements

Functions associated with coordination of simple and complex voluntary movements, performing movements in an orderly combination.

Inclusions: Right–left coordination, coordination of visually directed movements, such as eye–hand coordination and eye–foot coordination. Impairments such as dysdiadochokinesia.

Includes coordination functions: Finger-Nose Test and Heel-Knee-Shin Test

0 – No impairment. The person has no symptoms of coordination problems.

1 – Mild impairment. The person performs one of the tests inaccurately.
2 - Moderate impairment. The person performs two tests inaccurately or with intense tremor or ataxia.
3 - Severe impairment. The person performs all tests with intense tremor and marked ataxia.
4 - Complete impairment. The person cannot perform any of the tests.

b7650 Involuntary contractions of muscles

Functions of unintentional, nonpurposive or semipurposive involuntary contractions of a muscle or group of muscles, such as those involved as part of a psychological dysfunction.

Inclusions: Impairments such as choreatic and athetotic movements. Sleep-related movement disorders.

0 – No impairment. The person does not have involuntary movements.
1 - Mild impairment. The person rarely has involuntary movements (up to once a week).
2 – Moderate impairment. Involuntary movements are frequent, intensified by careful handling.
3 – Severe impairment. Involuntary movements are almost constant. The person can perform basic actions after adaptation.
4 - Complete impairment. The person cannot perform any activity due to persistent involuntary movements.

b7651 Tremor

Functions of alternating contraction and relaxation of a group of muscles around a joint, resulting in shakiness.

0- No impairment. No tremors present.
1- Mild impairment. Tremor is not noticeable at rest but occurs during voluntary movement and only during certain activities (e.g., writing, a specific work task).
2- Moderate impairment. The person has constant tremor and difficulty holding and manipulating small objects. Tremor intensifies with purposeful movement.
3- Severe impairment. The person has almost constant tremors and difficulties in posture, which interferes with daily activities.
4- Complete impairment. The person cannot perform any activity due to persistent tremor.

b770 Gait pattern functions.

Functions of movement patterns associated with walking, running, or other whole-body movements.

Inclusions: Walking patterns and running patterns. Impairments such as spastic gait, hemiplegic gait, paraplegic gait, asymmetric gait, limping, and stiff gait pattern.

0- No impairment. The person has correct and safe gait.

1- Mild impairment. The gait pattern is ataxic or sloppy but remains safe.
2- Moderate impairment. The gait is unsafe, asymmetric, and there is dragging of the leg or limping.
3- Severe impairment. The gait is unsafe, asymmetric, and reassigns weight. The person can move only a few steps.
4- Complete impairment. The person cannot move.

b780 Sensations related to muscles and movement functions

Sensations associated with the muscles or muscle groups of the body and their movement.

Inclusions: Sensations of muscle stiffness and tightness of muscles, muscle spasm or constriction, and heaviness of muscles.

b7801 Sensation of muscle spasm

Sensation of involuntary contraction of a muscle or a group of muscles.

0- No impairment. The person does not have sensations of muscle spasm.
1- Mild impairment. The muscle cramps occur irregularly.
2- Moderate impairment. The person has frequent episodes of muscle cramps that subside after exercise.
3- Severe impairment. The person has frequent episodes of muscle cramps that do not resolve after exercise and require medical treatment.
4- Complete impairment. The person has frequent and very troublesome episodes of muscle cramps, and medical treatment is ineffective.

BODY STRUCTURES

Body structures - s110 brain structures and s120 spinal cord and related structures were determined according to magnetic resonance imaging (MRI) findings.

s110 Brain structures

0- No impairments/changes.
1- Mild impairment: 1–2 demyelinating foci in the brain.
2- Moderate impairment: 3–8 demyelinating foci in the brain.
3- Severe impairment: multiple demyelinating foci in the brain.
4- Complete impairment: multiple demyelinating foci in the brain and brain atrophy.

s120 Spinal cord and related structures

0- No impairments/changes.
1- Mild impairment: 1 demyelinating foci in one segment.

2- Moderate impairment: 2 demyelinating foci in one segment.
3- Severe impairment: ≥ 3 demyelinating foci in one or several segments.
4- Complete impairment: multiple demyelinating foci in the spinal cord and spinal cord atrophy.

ACTIVITIES AND PARTICIPATION

Activity is the execution of a task or action by an individual. Participation is involvement in a life situation. The impairments are coded by a **performance** qualifier (what the person can perform at the present time in a real environment).

Performance is a construct that describes, as a qualifier, what individuals do in their current environment, demonstrating the person's involvement in life situations.

d110 Watching

Using the sense of seeing intentionally to experience visual stimuli, such as watching a sporting event or children playing.

0 - No difficulty. The person does not have problems watching.
1 - Mild difficulty. The person has mild impairment in understanding visual information.
2 - Moderate difficulty. The person has moderate impairment in understanding visual information.
3 - Severe difficulty. The person has severe impairment in understanding visual information.
4 - Complete difficulty. The person has complete impairment in understanding visual information.

d155 Acquiring skills

Developing basic and complex competencies in integrated sets of actions or tasks to initiate and follow through with the acquisition of a skill, such as manipulating tools or playing games such as chess.
Inclusion: Acquiring basic and complex skills.

0 - No difficulty. The person does not have problems with acquiring skills.
1 - Mild difficulty. The person has mild impairment in acquiring skills and performs activities, but it takes a long time.
2 - Moderate difficulty. The person has moderate impairment in acquiring skills and performs activities that are enjoyable.
3 - Severe difficulty. The person has severe impairment in acquiring skills but engages in activities with another person.

4 - Complete difficulty. The person has complete impairment in acquiring skills and does not perform any activities.

d160 Focusing attention

Intentionally focusing on specific stimuli, such as by filtering out distracting noises (i.e., ability to communicate on the phone when a television set is on).

0 - No difficulty. The person does not have problems with focusing attention.
1 - Mild difficulty. The person has slight disturbance and can focus attention briefly in the presence of extraneous noise.
2 - Moderate difficulty. The person has moderate disturbance and can focus attention when extraneous noise is silenced.
3 - Severe difficulty. The person has severe impairment, can focus attention when the extraneous noise is silenced and is assisted by another person.
4 - Complete difficulty. The person has complete impairment and is not engaged in any activity due to an inability to focus attention.

d163 Thinking

Formulating and manipulating ideas, concepts and images, whether goal-oriented or not, either alone or with others, such as creating fiction, proving a theorem, playing with ideas, brainstorming, meditating, pondering, speculating or reflecting.

0 - No difficulty. The person does not have problems with thinking.
1 – Mild difficulty. The person has mild impairments and purposefully forms all necessary thoughts, but it takes a long time.
2 - Moderate difficulty. The person has moderate impairments and forms the necessary thoughts, but it takes a long time to think. The person needs prompting and episodic help from another person.
3 - Severe difficulty. The person has severe impairments, forms and speaks sporadic thoughts. The person needs constant help from another person to formulate thoughts.
4 - Complete difficulty. The person has complete impairment and cannot engage in any thinking activities.

d166 Reading

Performing activities involved in the comprehension and interpretation of written language (e.g., books, instructions or newspapers in text or Braille) to obtain general knowledge or specific information.

0– No difficulty. The person does not have problems with reading.
1- Mild difficulty. The person has a mild reading disability. The person comprehends and interprets text, but it takes a long time.
2- Moderate difficulty. The person has a moderate reading disability. The person reads a text but takes a long time to comprehend and interpret and requires episodic help to perform reading tasks.
3- Severe difficulty. The person has a severe reading disability. The person reads a text but takes a long time to comprehend and interpret and requires constant help to perform reading tasks.
4- Complete difficulty. The person has complete reading impairment and does not read.

d170 Writing

Using or producing symbols or language to convey information, such as producing a written record of events or ideas or drafting a letter.

0- No difficulty. The person does not have problems with writing.
1- Mild difficulty. The person has mild writing impairments and takes a long time to write.
2- Moderate difficulty. The person has moderate writing impairments, takes a long time to write, and needs writing aids or episodic help.
3- Severe difficulty. The person has severe writing impairments. The person requires constant assistance from another person and an adapted environment.
4- Complete difficulty. The person has complete writing impairment and cannot write.

d175 Solving problems.

Finding solutions to questions or situations by identifying and analysing issues, developing options and solutions, evaluating potential effects of solutions, and executing a chosen solution, such as in resolving a dispute between two people. Includes solving both simple and complex problems.

0- No difficulty. The person is able independently to find solutions to simple and complex problems.
1- Mild difficulty. There is some lack of objectivity and appreciation of consequences, or more time is needed when independently finding solutions to simple and complex problems.
2- Moderate difficulty. The person can solve simple problems in terms of one question but cannot solve complex problems. The person needs episodic help to solve problems.
3- Severe difficulty. The person needs constant help from another person to solve problems.
4- Complete difficulty. The person cannot find problem-solving solutions.

d177 Making decisions

Choosing among options, implementing the choice, and evaluating the effects of the choice, such as selecting and purchasing a specific item or deciding to undertake and undertaking one task from among several tasks that need to be done.

0– No difficulty. There are no impairments in choosing and making decisions.
1 – Mild difficulty. There are minor impairments. The person chooses independently among several options, but it takes a long time to make a decision.
2- Moderate difficulty. There are moderate impairments. The person takes a long time to decide, and episodic help from another person is required to choose between several options and decide.
3- Severe difficulty. There is severe impairment. The person needs continuous support from another person to choose among multiple choices and make decisions.
4- Complete difficulty. The person cannot make decisions.

d210 Undertaking a single task
Conducting simple or complex and coordinated actions related to the mental and physical components of a single task, such as initiating a task, organizing time, space and materials for a task, pacing task performance, and conducting, completing and sustaining a task.
Inclusions: Undertaking a simple or complex task, undertaking a single task independently or in a group.
d2100 Undertaking a simple task
Preparing, initiating and arranging the time and space required for a simple task. Executing a simple task with a single major component, such as reading a book, writing a letter, or making one's bed.

0– No difficulty. The person independently undertakes and performs a simple task.
1- Mild difficulty. The person can perform a simple task independently, but it takes a long time.
2- Moderate difficulty. The person can undertake a simple task and perform it, but it takes a long time and requires occasional help from another person.
3- Severe difficulty. The person performs a simple task but requires constant help from another person.
4- Complete difficulty. The person cannot perform a simple task and is not independent.

d220 Undertaking multiple tasks
Conducting simple or complex and coordinated actions as components of multiple, integrated and complex tasks in sequence or simultaneously.
Inclusions: Undertaking multiple tasks, completing multiple tasks, undertaking multiple tasks independently and in a group.

d2201 Completing multiple tasks

Completing several tasks, together or sequentially

0– No difficulty. The person is independent in multitasking and completes tasks.

1- Mild difficulty. The person undertakes multiple tasks independently, but it takes a long time to complete.

2- Moderate difficulty. The person undertakes multiple tasks, but it takes a long time and requires episodic help from another person.

3- Severe difficulty. The person has severe difficulty completing multiple tasks and requires constant help from another person.

4- Complete difficulty. The person cannot complete tasks and is not independent.

d230 Carrying out daily routine

Conducting simple or complex and coordinated actions to plan, manage and complete the requirements of day-to-day procedures or duties, such as budgeting time and making plans for separate activities throughout the day.

Inclusions: Managing and completing the daily routine. Managing one's own activity level.

d2302 Completing the daily routine

Conducting simple or complex and coordinated actions to complete the requirements of day-to-day procedures or duties

0- No difficulty. The person independently plans, performs, and completes daily affairs.
1- Mild difficulty. There is some lack of objectivity and rationalism or more time is needed when independently planning, performing, and completing daily affairs.
2- Moderate difficulty. Daily routines are planned by others, but the person can perform the daily routines independently.
3- Severe difficulty. Daily routines are planned by others, and the person needs constant help when performing daily routines.
4- Complete difficulty. The person cannot plan, perform, and complete daily routines independently.

d240 Handling stress and other psychological demands

Conducting simple or complex and coordinated actions to manage and control the psychological demands required to perform tasks demanding significant responsibilities and involving stress, distraction or crises, such as driving a vehicle during heavy traffic or caring for multiple children.

Inclusions: Handling responsibilities and handling stress and crisis.

d2401 Handling stress

Conducting simple or complex and coordinated actions to cope with pressure, emergencies or stress associated with task performance

0- No difficulty. The person handles stressful situations independently and takes coordinated action.
1- Mild difficulty. The person handles stressful situations and takes concerted action but it takes a long time.
2- Moderate difficulty. The person has moderate difficulty handling stressful situations and problems. The person requires episodic help or 'hides it', expresses emotions that are incomprehensible to others, and often does not recognize problems.
3- Severe difficulty. The person has severe difficulty handling stressful situations and problems and requires constant help from others to handle stress.
4- Complete difficulty. The person cannot handle stress and is apathetic to problems.

d330 Speaking

Producing words, phrases and longer passages in spoken messages with literal and implied meaning, such as expressing a fact or telling a story orally.

0- No difficulty. The person speaks independently according to meaning and can tell long stories.
1- Mild difficulty. The person speaks independently by pronouncing words and phrases but has difficulties with longer passages and takes a long time to tell a story.
2- Moderate difficulty. The person speaks in short phrases, has difficulty expressing themselves and telling a long story, and requires episodic help from another person.
3- Severe difficulty. The person speaks but requires constant help from another person.
4- Complete difficulty. There are complete impairments. The person cannot speak.

d350 Conversation

Starting, sustaining and ending an interchange of thoughts and ideas carried out by means of spoken, written, sign or other forms of language, with one or more people one knows or who are strangers, in formal or casual settings.

Inclusions: starting, sustaining and ending a conversation. Conversing with one or many people

0- No difficulty. The person starts, maintains and ends the conversation independently.
1- Mild difficulty. The person starts, maintains, and finishes the conversation independently but takes a long time.
2- Moderate difficulty. The person starts a conversation but needs episodic help from another person to maintain and finish the conversation.
3- Severe difficulty. The person can only hold a conversation with the constant help of another person.
4- Complete difficulty. There are complete impairments. The person is not able to maintain a conversation.

d360 Using communication devices and techniques

Using devices, techniques and other means for the purposes of communicating, such as calling a friend on the telephone.

Inclusions: Using telecommunication devices, writing machines and communication techniques.

d3600 Using telecommunication devices

Using telephones and other machines, such as facsimile or telex machines, as a mean of communication.

0 - No difficulty. The person uses the phone freely.

1- Mild difficulty. The person uses the phone but takes a longer time to perform tasks or mild accessibility features.

2 - Moderate difficulty. The person uses the phone only if it is easy to use (i.e., large buttons, accessibility features) and with occasional help from another person.

3 - Severe difficulty. The person can use the phone with constant help from another person.

4 - Complete difficulty. The person cannot use the phone devices.

d410 Changing basic body position

Getting into and out of a body position and moving from one location to another, such as getting up out of a chair to lie down on a bed and getting into and out of positions of kneeling or squatting.

Inclusions: Changing body position from lying down, from squatting or kneeling, from sitting or standing, bending and shifting the body's centre of gravity.

d4103 Sitting

Getting into and out of a seated position and changing body position from sitting down to any other position, such as standing up or lying down.

Inclusions: Entering a sitting position with bent legs or cross-legged legs and getting into a sitting position.

0 – No difficulty. The person changes body position independently and safely.

1 – Mild difficulty. The person changes body position independently, but it takes a longer time.

2 – Moderate difficulties. The person changes body position with episodic support.

3 – Severe difficulty. The person changes body position with constant support or help from another person.

4 – Complete difficulty. The person cannot change body position and requires absolute assistance to change body position.

d415 Maintaining a body position

Staying in the same body position as needed, such as remaining seated or standing for work or school.

Inclusions: Maintaining a lying, squatting, kneeling, sitting and standing position.

d4154 Maintaining a standing position

Staying in a standing position for some time as needed, such as when standing in a queue. *Inclusions: Staying in a standing position on a slope, on slippery or hard surfaces.*

0 – No difficulty. The person maintains the correct standing position for the required time.
1 – Mild difficulty. The person stays upright for the required time but needs support.
2 – Moderate difficulty. The person maintains the required position with support for 10 min.
3 – Severe difficulty. The person maintains the position with support for 5 min.
4 – Complete difficulty. The person does not maintain standing body position.

d420 Transferring oneself

Moving from one surface to another, such as sliding along a bench or moving from a bed to a chair, without changing body position.

Inclusion: Transferring oneself while sitting or laying.

d4200 Transferring oneself while sitting

Moving from a sitting position on one seat to another seat on the same or a different level, such as moving from a chair to a bed.

Inclusions: Moving from a chair to another seat, such as a toilet seat or moving from a wheelchair to a car seat.

0 – No difficulty. The person stands up, sits down and transfers safely.
1 – Mild difficulty. The person is not fully independent - needs additional equipment (slippery board, parallel bars, special chairs, pillars, crutches, etc.
2 – Moderate difficulty. The person is not independent - requires additional aids (slippery board, parallel bars, special chairs, pillars, crutches, etc.) and the supervision of another person to prepare the conditions for the action (e.g., slip a slippery board under the buttocks, raise or lower a footrest).
3 – Severe difficulty. The person requires maximum contact assistance for the action (as shoulder support).
4 – Complete difficulty. The person requires absolute assistance.

d430 Lifting and carrying object

Raising up an object or taking something from one place to another, such as when lifting a cup or carrying a child from one room to another.

Inclusions: Lifting, carrying in the hands or arms, or on shoulders, hip, back or head and putting down.

d4301 Carrying in the hands (to hold a glass in the hand)

Taking or transporting an object from one place to another using the hands, such as when carrying a drinking glass.

0 – No difficulty. The hand grip (i.e., keeping a glass in the hand) is intact.
1 – Mild difficulty. The person holds a glass in the hand for a short time.
2 – Moderate difficulty. The person holds a glass in the hand when using assistive devices (compensatory technique).
3 – Severe difficulty. The person holds a glass in the hand using assistive devices (compensatory technique) and with constant help from another person.
4 – Complete difficulty. The person is not able to perform the action.

d440 Fine hand use
Performing the coordinated actions of handling objects, picking up, manipulating and releasing them using one's hand, fingers and thumb, such as lifting coins off a table or turning a dial or knob.
Inclusions: Picking up, grasping, manipulating and releasing,
d4400 Picking up
Lifting or picking up a small object with hands and fingers, such as a pencil.

0 – No difficulty. The person grasps and picks up small objects with their fingers.
1 – Mild difficulty. The person picks up and lifts small objects with fingers, but not always successfully.
2 – Moderate difficulty. The person picks up but does not lift small objects with their fingers.
3 – Severe difficulty. The person picks up small objects with their hand or fingers with the constant use of aids.
4 – Complete difficulty. The person cannot manipulate objects with their hands or fingers.

d445 Hand and arm use
Performing the coordinated actions required to move objects or to manipulate them by using hands and arms, such as when turning door handles or throwing or catching an object.
Inclusions: Pulling or pushing objects, reaching, turning or twisting the hands or arms, throwing and catching.
d4454 Throwing
Using fingers, hands and arms to lift something and propel it with some force through the air, such as when tossing a ball.
d4455 Catching

Using fingers, hands and arms to grasp a moving object to bring it to a stop and hold it, such as when catching a ball.

0 – No difficulty. The person throws or catches the ball.

1 – Mild difficulty. The person throws or catches the ball, but not always accurately.

2 – Moderate difficulty. The person throws or catches the ball independently only when the position is comfortable and the ball is within easy reach.

3 - Severe difficulty. The person is only able to throw or catch the ball with help of another person.

4 - Complete difficulty. The person does not perform throwing or catching action.

d450 Walking.

Moving along a surface on foot, step by step, so that one foot is always on the ground, such as when strolling, sauntering, walking forwards, backwards, or sideways.

Inclusions: Walking short or long distances, walking on different surfaces, and walking around obstacles.

0- No difficulties when walking on different surfaces with no danger for themselves or surrounding people, and the person realizes the meaning of the action, the direction, and the final task.
1- Mild difficulty. The person walks (moves) independently, but it takes a longer time. The obstacles are overcome, but it takes a longer time. An aid (stick, crutches, walker, wheelchair, etc.) is used sometimes but without aid from other persons.
2- Moderate difficulty. The person walks (moves) independently, but it takes a long time, and walking is unsafe. There is a risk for falls, causing an emergency situation. There is a constant need for aid (stick, crutches, walker, wheelchair, etc.) and episodic aid from another person (verbal or physical) to overcome obstacles.
3- Severe difficulty. The person is not able to walk independently and safely. There is a constant need for aid (stick, crutches, walker, wheelchair, etc.) and constant aid from another person.
4- Complete difficulty. The person cannot walk.

d4500 Walking short distances

Walking for less than a kilometre, such as walking around rooms or hallways, within a building or for short distances outside.

d4501 Walking long distances

Walking for more than a kilometre, such as across a village or town, between villages or across open areas.

0- No difficulties when walking on different surfaces with no danger for themselves or surrounding people, and the person realizes the meaning of the action, the direction, and the final task.

1- Mild difficulty. The person walks (moves) independently, but it takes a longer time. The obstacles are overcome, but it takes a longer time. An aid (stick, crutches, walker, wheelchair, etc.) is used sometimes but without aid from other persons.
2- Moderate difficulty. The person walks (moves) independently, but it takes a long time, and walking is unsafe. There is a risk of falls, causing emergencies. There is a constant need for aids (e.g., a stick, crutches, a walker, or a wheelchair) and episodic help from another person (verbal or physical) to overcome obstacles.
3- Severe difficulty. The person cannot walk independently and safely. There is a constant need for aids (e.g., a stick, crutches, a walker, or a wheelchair) and constant help from another person.
4- Complete difficulty. The person cannot walk.

d455 Moving around

Moving the whole body from one place to another by means other than walking, such as climbing over a rock or running down a street, skipping, scampering, jumping, somersaulting or running around obstacles.

Inclusions: Crawling, climbing, running, jogging, jumping and swimming.

0- No difficulties. The person is able to move around independently and can climb and run up and down without aid. Movement is performed safely and consciously.

1- Mild difficulties. The person is almost independent but needs aid when climbing or running up and down; needs handrails, sticks, or other aids to guarantee safety, but no aid from surrounding persons. It takes longer time to move.

2- Moderate difficulties. The person needs contact support, independently performs 75% of movement, and needs random (short) contact support to maintain and stabilize balance.

3- Severe difficulties. The person performs 25–50% of movement when climbing or running a short distance and needs contact support from other persons.

4- Complete difficulties. The person needs absolute support to climb or run.

d4552 Running

Moving with quick steps so that both feet may be simultaneously off the ground.

0- No difficulties. The person is completely autonomous during the activity and can run without any additional aids or holding on. The person performs the activity safely (without endangering self and/or others).
1- Mild difficulties. The person is almost independent in the activities - runs with the occasional need for a handrail, stick or other mobile support or safety device, without needing another person nearby. It takes a long time to perform the action.

2- Moderate difficulties. The person needs contact assistance, and performs 75% of the action but needs occasional (short-term) assistance from holding to stabilize balance and occasionally needs assistance from another person.
3- Severe difficulties. The person performs 25–50% of the action but runs only a short distance and needs constant assistance from another person.
4- Complete difficulties. The person cannot run.

d460 Moving around in different locations

Walking and moving around in various places and situations, such as walking between rooms in a house, within a building or down the street of a town.

Inclusions: Moving around within the home, crawling or climbing within the home, walking or moving within buildings other than the home and outside the home and other buildings.

d4600 Moving around within the home

Walking and moving around in one's home, within a room, between rooms, and around the whole residence or living area. *Inclusions: Moving from floor to floor, on an attached balcony, courtyard, porch or garden.*

0- No difficulties. The person moves safely without using compensatory walking/moving aids.
1- Mild difficulties. The person moves safely with a compensatory walking/movement aid.
2- Moderate difficulties. The person moves unsafely with (non)compensatory walking/movement aid.
3- Severe difficulties. The person can get from one place to another if there are no thresholds.
4- Complete difficulties. The person does not move.

d4602 Moving around outside the home and other buildings

Walking and moving around close to or far from one's home and other buildings, without the use of transportation, public or private, such as walking for short or long distances around a town or village.

Inclusions: Walking or moving down streets in the neighbourhood, town, village or city, and moving between cities.

0 - No difficulties. The person moves freely around different areas, both inside and outside the building, regardless of height differences and stairs.

1 – Mild difficulties. The person moves around different places but cannot cover distances between buildings if they are not close together, and has difficulty climbing stairs, especially if there are no handrails.

2 - Moderate difficulties. The person moves inside the building without any problems but cannot move safely outside the buildings.

3 – Severe difficulties. The person only moves inside the building, does not climb stairs, and can move over small thresholds between rooms.

4 - Complete difficulties. The person does not walk/move either inside or outside the building.

d465 Moving around using equipment

Moving the whole body from place to place, on any surface or space, by using specific devices designed to facilitate moving or create other ways of moving around, such as with skates, skis, or scuba equipment, or moving down the street in a wheelchair or a walker

Inclusions: Mobility with assistive devices (e.g., a wheelchair, a low/high walker, or crutches).

0 – No difficulties. The person moves independently with a wheelchair, walker, cane, or crutch from one place to another on different surfaces.

1 – Mild difficulties. The person moves independently with a wheelchair, walker, cane, or crutch from one place to another on level surfaces.

2 – Moderate difficulties. The person moves independently with a wheelchair, walker, wand, or crutch from one place to another on a level surface, but with rest, and it takes a long time.

3 – Severe difficulties. The person moves with a wheelchair, walker, walking stick, or crutch from one place to another on a level surface but with the assistance of another person.

4 – Complete difficulties. The person cannot perform the action.

9- the person does not use assistive devices when moving.

d470 Using transportation

Using transportation to move around as a passenger, such as being driven in a car or on a bus, rickshaw, jitney, animal-powered vehicle, or private or public taxi, bus, train, tram, subway, boat or aircraft.

Inclusions: Using human-powered transportation and using private motorized or public transportation.

d4702 Using public motorized transportation

Being transported as a passenger by a motorized vehicle over land, sea or air designed for public transportation, such as being a passenger on a bus, train, subway or aircraft

0 – No difficulties. The person uses transport independently.

1 - Mild difficulties. The person uses transport independently, accessed by mobility aids.

2 - Moderate difficulties. The person uses transport, accessed by mobility aids, but with rest or with occasional assistance from another person.

3 - Severe difficulties. The person does not use transport independently. The person needs constant assistance from another person.

4 - Complete difficulties. The person cannot use transport.

d475 Driving

Being in control of and moving a vehicle or the animal that draws it, travelling under one's own direction or having at one's disposal any form of transportation, such as a car, bicycle, boat or animal-powered vehicle.

Inclusions: Driving human-powered transportation, motorized vehicles, and animal-powered vehicles.

d4751 Driving motorized vehicles

Driving a vehicle with a motor, such as an automobile, motorcycle, motorboat or aircraft.

0– No difficulties. The person can drive without restrictions.

1- Mild difficulties. The person can drive but with difficulty navigating.

2- Moderate difficulties. The person can drive but with difficulty navigating and operates a vehicle only in familiar routes/surroundings.

3- Severe difficulties. The person cannot operate a vehicle without special driving aids – another person, special driving aid equipment, or special vehicle access equipment (i.e., wheelchair access).

4- Complete difficulties. The person cannot drive due to illness.

9 – the person has never obtained driving skills and does not have a driving licence.

d510 Washing oneself

Washing and drying one's whole body or body parts, using water and appropriate cleaning and drying materials or methods, such as bathing, showering, washing hands and feet, face and hair, and drying with a towel.

Inclusions: Washing body parts, the whole body. and drying oneself.

d5101 Washing whole body

Applying water, soap and other substances to the whole body to clean oneself, such as taking a bath or shower.

0 - No difficulties. The person bathes independently (soaping, rubbing and washing the body), and wipes themselves. The person washes safely.

1 – Mild difficulties. Bathing and wiping are independent, but it requires an adapted environment and/or prostheses and may require verbal assistance (prompting, telling the person what to do) and/or preparation of the bath and washing materials (cleaning the bath, adding water). It takes a longer time than for a healthy person and is not fully safe.

2 – Moderate difficulties. The person requires minimal contact assistance (e.g., rubbing one or two areas (legs, feet) with a washcloth)) and aids (may need help wiping back, legs, and affected body parts).

3 – Severe difficulty. Bathing and wiping require contact assistance for all activities.

4 - Complete difficulties. The person does not participate in the bathing process.

d520 Caring for body parts

Looking after the parts of the body, such as skin, face, teeth, scalp, nails and genitals, that require more than washing and drying.

Inclusions: Caring for the skin, teeth, hair, finger and toenails.

d5204 Caring for toenails

Cleaning, trimming or polishing the nails of the toes.

0 - No difficulties. The person can conduct self-care for toenails and does it safely.
1 – Mild difficulties. The self-care of toenails is independent, but it takes longer than for a healthy person and is not safe enough.
2 – Moderate difficulties. The independent care for toenails requires an adapted environment and/or prostheses/orthotics. The person might need verbal support (prompting, action instructions).
3 – Severe difficulties. The person needs contact assistance with toenail care.
4 - Complete difficulties. The person cannot care for their body parts.

d530 Toileting

Planning and Conducting the elimination of human waste (menstruation, urination and defecation) and cleaning oneself afterwards.

Inclusions: regulating urination, defecation and menstrual care

0 - No difficulties. The person uses the toilet independently and does it safely.
1 – Mild difficulties. The person uses the toilet independently but it is not safe enough and is performed slowly at a low level of hygiene.
2 – Moderate difficulties. The person uses the toilet with minimal assistance. Individually, the person is not safe enough and performs at a lower level of hygiene, with occasional instances of not cleaning.
3 – Severe difficulties. The person uses the toilet with the constant help of another person.
4 – Complete difficulties. The person cannot take care of toileting.

d540 Dressing

Conducting the coordinated actions and tasks of putting on and taking off clothes and footwear in sequence and in keeping with climatic and social conditions, such as by putting on, adjusting and removing shirts,

skirts, blouses, pants, undergarments, saris, kimono, tights, hats, gloves, coats, shoes, boots, sandals and slippers.
Inclusions: Putting on or taking off clothes and footwear and choosing appropriate clothing.

0 - No difficulties. The person dresses and undresses independently, chooses appropriate clothing and does so safely.
1 - Mild difficulties. The person dresses and undresses independently and chooses appropriate clothing, but it takes a long time and is not safe enough.
2 – Moderate difficulties. The person uses prostheses/orthotics to get ready or needs minimal contact assistance (e.g., to start the dressing action or help with small items of clothing (buttons, pins, buckles, and laces) or sometimes to advise on appropriate clothing, outline the steps of getting ready, and/or to urge the person to prepare.
3 - Severe difficulties. The person needs contact help from another person to dress and undress and does not choose appropriate clothing independently.
4 - Complete difficulties. The person does not participate in the preparation process and needs to be fully clothed by others.

d550 Eating
Conducting the coordinated tasks and actions of eating food that has been served, bringing it to the mouth and consuming it in culturally acceptable ways, cutting or breaking food into pieces, opening bottles and cans, using eating implements, having meals, feasting or dining.

0 - No difficulties. The person eats independently (chopping food, opening dishes, and using cutlery; however, this does not include food preparation) and does so safely.
1 – Mild difficulties. The person eats independently but takes longer than healthy individuals and is not safe enough. The person may need verbal help (prompting and advice) and/or preparation (e.g., cutting meat, spreading butter on bread, or pouring a drink).
2 – Moderate difficulties. The person uses dentures/orthotics and needs minimal contact assistance (e.g., handing a tool, occasionally pushing a piece of food onto a spoon or fork).
3 - Severe difficulties. The person requires medium/maximal contact assistance for all activities when eating (e.g., holding tools and/or placing food directly in the mouth at all times).
4 - Complete difficulties. The person needs to be fed without their involvement.

d560 Drinking

Taking hold of a drink, bringing it to the mouth, consuming the drink in culturally acceptable ways, mixing, stirring and pouring liquids for drinking, opening bottles and cans, drinking through a straw or drinking running water such as from a tap or a spring, and feeding from the breast.

0 - No difficulties. The person drinks from a cup or glass independently and safely.
1 – Mild difficulties. The person drinks from a cup or glass but has difficulties opening a container.
2 – Moderate difficulties. The person drinks from a cup with moderate help.
3 – Severe difficulties. The person drinks only from a cup with two handles, does not open the container and needs more than moderate help.
4 – Complete difficulties. The person does not drink independently and needs to be hydrated.

d570 Looking after one's health
Ensuring physical comfort, health and physical and mental well-being, such as by maintaining a balanced diet and an appropriate level of physical activity, keeping warm or cool, avoiding harm to health, following safe sex practices, including using condoms, receiving immunizations and regular physical examinations.
Inclusions: Ensuring one's physical comfort. Managing diet and fitness. Maintaining one's health.

0 - No difficulties. The person takes care of their personal health and health maintenance.
1 - Mild difficulties. The person takes care of their personal health independently, and only verbal aids are needed (reminders and encouragement).
2 - Moderate difficulties. The person takes care of their personal health when the health care action plan is drawn up by others and needs constant supervision.
3 - Severe difficulty. The person needs contact support for health care.
4 - Complete difficulties. The person does not take care of their personal health.

d620 Acquisition of goods and services
Selecting, procuring and transporting all goods and services required for daily living, such as selecting, procuring, transporting and storing food, drink, clothing, cleaning materials, fuel, household items, utensils, cooking ware, domestic appliances and tools. Procuring utilities and other household services.
Inclusions: Shopping and gathering daily necessities.
d6200 Shopping
Obtaining, in exchange for money, goods and services required for daily living (including instructing and supervising an intermediary to do the shopping), such as selecting food, drink, cleaning materials, household

items or clothing in a shop or market, comparing the quality and price of the items needed, negotiating and paying for selected goods or services, and transporting goods.

0 - No difficulties. The person is self-sufficient in the purchase of goods and services.
1- Mild difficulties. The person independently acquires goods and services but takes a long time.
2- Moderate difficulties. The person may need contact assistance in one of the three activities (selecting, purchasing or delivering goods).
3- Severe difficulties. The person needs contact assistance in all three activities (selecting, purchasing or delivering goods).
4- Complete difficulties. The person cannot acquire goods and services.

d630 Preparing meals
Planning, organizing, cooking and serving simple and complex meals for oneself and others, such as by making a menu, selecting edible food and drink, gathering ingredients for preparing meals, cooking with heat and preparing cold foods and drinks, and serving the food.
Inclusions: Preparing simple and complex meals.
d6300 Preparing simple meals
Organizing, cooking and serving meals with a small number of ingredients that require easy preparation and serving methods, such as making a snack or small meal, and transforming food ingredients such as rice or potatoes by cutting and stirring, boiling and heating.

0 - No difficulties. The person plans and prepares simple meals independently, serves themselves and others, and does so safely.
1 – Mild difficulties. The person independently prepares simple meals, serves self and others, but takes longer than healthy, may need supervision to ensure safety and/or uses adapted environment and/or prosthesis, may need verbal support (prompting, directions).
2 – Moderate difficulties. The person needs minimal contact assistance (e.g., handing out tools, putting on orthoses/prostheses, planning menus, and outlining cooking steps).
3 – Severe difficulties. The person does not cook the meal independently but can perform individual elements of the meal preparation (e.g., cutting and peeling with or without orthoses/prostheses, verbal commands). The main work is done by another person.
4 - Complete difficulties. The person is not involved in the food preparation process.

d640 Doing housework

Managing a household by cleaning the house, washing clothes, using household appliances, storing food and disposing of garbage, such as by sweeping, mopping, washing counters, walls and other surfaces. Collecting and disposing of household garbage. Tidying rooms, closets and drawers. Collecting, washing, drying, folding and ironing clothes. Cleaning footwear. Using brooms, brushes and vacuum cleaners. Using washing machines, driers and irons.

Inclusions: Washing and drying clothes and garments. Cleaning cooking area and utensils. Cleaning the living area. Using household appliances. Storing daily necessities and disposing of garbage.

d6402 Cleaning living area

Cleaning the living areas of the household, such as by tidying and dusting, sweeping, swabbing, mopping floors, cleaning windows and walls, cleaning bathrooms and toilets, cleaning household furnishings.

0 – No difficulties. The person cleans the living space independently, and collects and removes rubbish.
1 - Mild difficulties. The person performs household activities independently but takes longer time than a healthy person.
2 - Moderate difficulty. The person does not plan cleaning activities in the living area, takes longer than an able-bodied person to complete activities, and needs verbal support in the form of advice and guidance.
3 - Severe difficulties. The person partially cleans the living area with the help of other contacts.
4 - Complete difficulties. The person does not conduct household activities.

d650 Caring for household objects

Maintaining and repairing household and other personal objects, including the house and its contents, clothes, vehicles and assistive devices, and caring for plants and animals, such as painting or wallpapering rooms, fixing furniture, repairing plumbing, ensuring the proper working order of vehicles, watering plants, grooming and feeding pets and domestic animals.

Inclusions: Making and repairing clothes, maintaining dwellings, furnishings and domestic appliances. Maintaining vehicles. Maintaining assistive devices. Taking care of plants (indoor and outdoor) and animals.

0 - No difficulties. The person carries for household objects independently.
1 - Mild difficulties. The person carries for household objects independently but takes longer than a healthy person.
2 - Moderate difficulty. The person does not plan to care for household objects, takes longer than an able-bodied person to complete activities, and needs verbal support in the form of advice and guidance.
3 - Severe difficulties. The person partially carries for household objects with the help of other contacts.
4 - Complete difficulties. The person does not carry for household objects.

d660 Assisting others

Assisting household members and others with their learning, communicating, self-care, movement, within the house or outside. Being concerned about the well-being of household members and others.

Inclusions: Assisting others with self-care, movement, communication, interpersonal relations, nutrition and health maintenance.

0 - No difficulties. The person actively helps others in areas that are important to them (e.g., education, mobility, self-care, communication).
1 - Mild difficulties. The person actively helps others but cannot help in one of the domains.
2 - Moderate difficulties. The person actively helps others in areas that do not require physical work.
3 - Severe difficulties. The person can provide help if others are involved.
4 - Complete difficulties. The person does not help others.

d710 Basic interpersonal interactions

Interacting with people in a contextually and socially appropriate manner, such as by showing consideration and esteem when appropriate or responding to the feelings of others.

Inclusions: Showing respect, warmth, appreciation, and tolerance in relationships. Responding to criticism and social cues in relationships. Using appropriate physical contact in relationships.

0 - No difficulty. Basic interpersonal interactions and communication is not impaired. The person actively shows attention and responds to others' feelings.
1 - Mild difficulty. The person actively shows attention and responds to others' feelings, interacting only within close circles and/or with loved ones.
2 - Moderate difficulty. The person shows attention and responds to others' feelings when communication is assisted by others.
3 - Severe difficulty. The person can show attention and react to others' feelings when reacting to others.
4 - Complete difficulties. The person does not show attention and does not react to the feelings of others.

d720 Complex interpersonal interactions

Maintaining and managing interactions with other people in a contextually and socially appropriate manner, such as by regulating emotions and impulses, controlling verbal and physical aggression, acting independently in social interactions and acting in accordance with social rules and conventions.

Inclusions: Forming and terminating relationships. Regulating behaviours within interactions. Interacting according to social rules and maintaining social space.

d7200 Forming relationships

Beginning and maintaining interactions with others for a short or long period, in a contextually and socially appropriate manner, such as by introducing oneself, finding and establishing friendships and professional relationships, starting a relationship that may become permanent, romantic or intimate

0– No difficulties. The function is not impaired.
1- Mild difficulties. The person avoids making new contacts.
2- Moderate difficulties. The person avoids making new contacts and developing friendship relationships. Encouragement is needed.
3- Severe difficulties. The person makes new contacts and develops friendships only with the help of others.
4- Complete difficulties. The person does not make new contacts or develop friendship relationships.

d750 Informal social relationships

Entering into relationships with others, such as casual relationships with people living in the same community or residence or with coworkers, students, playmates or people with similar backgrounds or professions.

Inclusions: Informal relationships with friends, neighbours, acquaintances, coinhabitants and peers.

d7501 Informal relationships with neighbours

Creating and maintaining informal relationships with people who live in nearby dwellings or living areas.

0- No difficulties. The person builds and maintains good relations with neighbours.
1- Mild difficulties. The person builds and maintains relationships with neighbours, and the relationship is rather formal.
2- Moderate difficulties. The person builds and maintains good relations with neighbours when the person feels the neighbours’ support and willingness to interact.
3- Severe difficulties. The person maintains relations with neighbours only with the help of the household.
4- Complete difficulties. The person does not maintain relations with neighbours.

d760 Family relationships.

Creating and maintaining kinship relationships, such as with members of the nuclear family, extended family, foster and adopted family and step-relationships, more distant relationships such as second cousins, or legal guardians.

Inclusions: Parent–child and child–parent relationships and sibling and extended family relationships.

0- No difficulties. The person creates and maintains kinship relationships with immediate and extended family.
1- Mild difficulties. The person creates and maintains kinship relationships with the immediate family and satisfying relationships with the extended family.
2- Moderate difficulties. The person creates and maintains kinship relationships with the immediate family but not with the extended family.
3- Severe difficulties. The person creates and maintains a relationship with the immediate family with support from the family and on their own desire to communicate.
4- Complete difficulties. The person is not concerned about creating and maintaining family relationships.

d770 Intimate relationships

Creating and maintaining close or romantic relationships between individuals, such as husbands and wives, lovers or sexual partners.

Inclusions: Romantic, spousal and sexual relationships.

d7701 Spousal relationships

Creating and maintaining an intimate relationship of a legal nature with another person, such as in a legal marriage, including becoming and being a legally married wife or husband or an unmarried spouse

0– No difficulties. The person has a family and spousal relations are good.
1- Mild difficulties. The spousal relationship is supportive, but there are problems.
2- Moderate difficulty, a spouse is deceased.
3- Severe difficulties. The person is divorced/split.
4- Complete difficulties. The person is single without their own family.

d825 Vocational training

Engaging in all activities of a vocational programme and learning the curriculum material in preparation for employment in a trade, job or profession.

0– No difficulties. The person completed vocational training with a view to gain employment.
1- Mild difficulties. The person completed vocational training with no intention of becoming employed.

2- Moderate difficulties. The person did not complete vocational training due to a lack of intention to become employed.
3- Severe difficulties. The person did not complete vocational training due to illness.
4- Complete difficulties. The person did not participate in vocational training due to illness.

d830 Higher education
Engaging in the activities of advanced educational programmes in universities, colleges and professional schools and learning all aspects of the curriculum required for degrees, diplomas, certificates and other accreditations, such as completing a university bachelor's or master's course of study, medical school or other professional school.

0– No difficulties. The person completed higher education with a view to gain employment.
1- Mild difficulties. The person completed tertiary education with no intention of entering employment.
2- Moderate difficulties. The person did not complete tertiary education with no intention of employment.
3- Severe difficulties. The person did not complete higher education due to illness.
4- Complete difficulties. The person is not enrolled in tertiary education due to illness**.**

d845 Acquiring, keeping and terminating a job
Seeking, finding and choosing employment; being hired and accepting employment; maintaining and advancing through a job, trade, occupation or profession; and leaving a job in an appropriate manner.
Inclusions: Seeking employment, preparing a resume or curriculum vitae, contacting employers and preparing for interviews, maintaining a job, monitoring one's own work performance, giving notice, and terminating a job.
d8451 Maintaining a job
Performing job-related tasks to keep an occupation, trade, profession or other form of employment, and obtaining promotion and other advancements in employment.
0– No difficulties. The person is working at a job that guarantees not only survival but also professional and other development.
1- Mild difficulties. The person works at a job that guarantees survival but not professional or other advancements.
2- Moderate difficulties. The person works at a job that guarantees survival and professional and other development with the help of a personal assistant.
3- Severe difficulties. The person works at a job that guarantees survival only with the help of a personal assistant.

4- Complete difficulties. The person has lost work capacity.

d850 Remunerative employment.

Engaging in all aspects of work as an occupation, trade, profession, or other form of employment for payment as an employee, full- or part-time, or self-employed, such as seeking employment and getting a job, doing the required tasks of the job, attending work on time as needed, supervising other workers or being supervised, and performing required tasks alone or in groups.

Inclusions: Self-employment, part-time and full-time employment.

0- No difficulties. There are no difficulties with paid work.
1- Mild difficulties. The person works independently but needs occasional help (for example, to find work and get employment).
2- Moderate difficulties. The person works independently but needs occasional help (for example, to perform complex tasks), works a shorter time, and performs customized work or works in a customized environment.
3- Severe difficulties. The person can perform paid work but needs continuous supervision and help from other persons.
4- Complete difficulties. The person cannot perform paid work.

d8501 Part-time employment

Engaging in all aspects of work for payment on a part-time basis, such as seeking employment and getting a job, doing the tasks required of the job, attending work on time as needed, supervising other workers or being supervised, and performing required tasks alone or in groups.

0– No difficulties. There are no difficulties in part-time paid work.

1- Mild difficulties. The person is employed in a partly paid job but needs occasional help from another person (e.g., to find a job and get a job).

2- Moderate difficulty: the person is employed in a partially paid job but needs occasional support from another person (e.g., for more complex tasks) and works in an adapted working environment.

3- Severe difficulties. The person cannot work independently in a partially paid job and always needs another person to supervise and assist on a regular basis.

4- Complete difficulties. The person cannot work in part-time paid work.

d8502 Full-time employment

Engaging in all aspects of work for payment on a full-time basis, such as seeking employment and getting a job, doing the required tasks of the job, attending work on time as needed, supervising other workers or being supervised, and performing required tasks alone or in groups.

0– No difficulties. There are no difficulties in getting and performing in a full-time paid job.
1- Mild difficulties. The person is employed in a full-time paid job but needs occasional help from another person (e.g., to find a job and get a job).
2- Moderate difficulties. The person is employed in a full-time paid job but needs the occasional support of another person/assistant (e.g., for more complex tasks) and is working in an adapted working environment.
3- Severe difficulties. The person cannot work independently in a full-time paid job and always needs another person to supervise and assist at all times.
4- Complete difficulties. The person is not in full-time paid work.

d860 Basic economic transactions

Engaging in any form of simple economic transaction, such as using money to purchase food or bartering, exchanging goods or services, or saving money.

0– No difficulties. There are no difficulties in participating in simple economic interactions.
1- Mild difficulties. The person has no major difficulties in participating in simple economic interactions but has occasional difficulty evaluating purchasing and saving decisions.
2- Moderate difficulties. The person has difficulties in simple economic interactions and requires occasional support from another person to budget and carry out economic transactions.
3- Severe difficulties. The person has difficulties in simple economic interactions and needs constant help from another person to participate in economic transactions.
4- Complete difficulties. The person does not participate in simple economic interactions.

d870 Economic self-sufficiency

Having command over economic resources from private or public sources to ensure economic security for present and future needs.

Inclusions: Personal economic resources and public economic entitlements.

d8700 Personal economic resources

Having command over personal or private economic resources to ensure economic security for present and future needs.

0– No difficulties. The person does not have economic difficulties and has present and future economic security.
1- Mild difficulties. The person secures present personal economic security but may have difficulties planning and budgeting future resources.
2- Moderate difficulties. The person requires occasional support from another person to ensure economic security.
3- Severe difficulties. The person requires ongoing help from another person to achieve economic security.
4- Complete difficulties. The person cannot achieve economic security.

d910 Community life
Engaging in all aspects of community social life, such as engaging in charitable organizations, service clubs or professional social organizations.
Inclusions: Informal and formal associations. Ceremonies.
d9101 Formal associations
Engaging in professional or other exclusive social groups, such as associations of lawyers, physicians or academics.

0– No difficulties. The person has no difficulties and actively participates in organizations and unions.
1- Mild difficulties. The person participates in organizations and unions episodically or passively.
2- Moderate difficulties. The person participates in organizations and unions with occasional help, encouragement, or invitation from another person.
3- Severe difficulties. The person participates in organizations and unions only with the constant help of another person.
4- Complete difficulties. The person does not participate in organizations or unions

d920 Recreation and leisure
Engaging in any form of play, recreational or leisure activity, such as informal or organized play and sports, programmes of physical fitness, relaxation, amusement or diversion, going to art galleries, museums, cinemas or theatres. Engaging in crafts or hobbies, reading for enjoyment, playing musical instruments. sightseeing, tourism and travelling for pleasure.
Inclusions: Play, sports, arts and culture, crafts, hobbies and socializing.

0 - No difficulties. The person organizes and spends their leisure time independently.

1 - Mild difficulties. The person needs to be encouraged to participate in activities but can organize their leisure time independently.
2 - Moderate difficulties. The person cannot organize their leisure time, needs to be encouraged to participate in the activity, and needs ongoing support from another person.
3 - Severe difficulties. The person cannot organize their leisure activities and only participates with ongoing support from another person.
4 - Complete difficulties. The person is passive (not doing anything, just laying or sitting) in their leisure time.

d930 Religion and spirituality
Engaging in religious or spiritual activities, organizations and practices for self-fulfilment, finding meaning, religious or spiritual value and establishing connection with a divine power, such as attending a church, temple, mosque or synagogue, praying or chanting for a religious purpose, and spiritual contemplation.
Inclusions: Organized religion and spirituality.

0 - No difficulties. The person participates and engages in the ceremony independently.
1 - Mild difficulties. The person participates episodically and independently in the rites.
2 - Moderate difficulties. The person can participate and engage in the rite with the occasional help of another person.
3 - Severe difficulties. The person cannot participate and engage in the rite and requires ongoing support from another person.
4 - Complete difficulties. The person cannot participate and engage in the rite due to illness.
ENVIRONMENTAL FACTORS: SUPPORT, RELATIONSHIPS, AND ATTITUDES.
e1101 Drugs
Any natural or human-made object or substance gathered, processed or manufactured for medicinal purposes, such as allopathic and naturopathic medication.

0 - No barrier or barrier is very low: 0–4%.
-1 - Mild barrier: 5–24% negative impact of medication, but can always be prevented with a visit to the doctor, and medication will be prescribed.
-2 - Moderate barrier: 25–49% negative impact of medication, but can be prevented by receiving medical care and following medical recommendations.
-3 - Severe barrier: 50–95% negative impact of medication and cannot be prevented by receiving medical care; experiencing medication side effects.

-4 - Complete barrier: 96–100% negative impact of medication that cannot be prevented or no medication is available.

0 - No or very low facilitator: 0–4%.

+1 - Mild facilitator: 5–24% positive effect of medication, onset of treatment.

+2 - Moderate facilitator: 25–49% positive effect of medication, treatment started, unrecognized effect of medication.

+3 - Substantial facilitator: 50–95% positive effect of medication; inconsistently feels the positive effect of medication.

+4 - Complete facilitator: 96–100% positive effect of medication; consistently feels the positive effect of medication.

e115 Products and technology for personal use in daily living

Equipment, products and technologies used by people in daily activities, including those adapted or specially designed, in, on or near the person using them.

Inclusions: General and assistive products and technology for personal use.

0 - No or very low barrier: 0–4%.

-1 - Mild barrier: 5–24% negative impact, 5–24% problem, but always avoidable, must purchase a product for personal use in daily living.

-2 – Moderate barrier: 25–49% negative impact, but can be prevented by prescribing or referring to products for daily personal use.

-3 – Severe barrier: 50–95% negative impact, negative problem due to poor quality of the product meant for daily personal use.

-4 - Complete barrier: 96–100% negative impact, negative problem and cannot be prevented, as there is no suitable product for daily personal use or it is unavailable.

0 - No or very low facilitator: 0–4%.

+1 - Mild facilitator: 5–24% positive effect of product for daily personal use, but product access is variable, its effect is inconsistent or the product does not facilitate all functions.

+2 - Moderate facilitator: 25–49% positive effect of product for daily personal use; product is available, but the effect is not fully consistent and it does not facilitate all functions.

+3 - Substantial facilitator: 50–95% positive effect of product for daily personal use; product is available, its effect is consistent, the quality is good, and it helps facilitate most functions.

+4 - Complete facilitator: 96–100% positive effect of product for daily personal use; product is available, the effect is consistent, the quality is high, and it helps facilitate all functions.

e120 Products and technology for personal indoor and outdoor mobility and transportation
Equipment, products and technologies used by people in activities of moving inside and outside buildings, including those adapted or specially designed, in, on or near the person using them.
Inclusions: General and assistive products and technology for personal indoor and outdoor mobility and transportation.

0 – No or very low barrier: 0–4%.
-1 - Mild barrier: 5–24% negative impact, 5–24% problem, but always avoidable, must purchase a product for personal indoor and outdoor mobility and transportation.
-2 – Moderate barrier: 25–49% negative impact, but can be prevented by prescribing or referring to products for personal indoor and outdoor mobility and transportation.
-3 – Severe barrier: 50–95% negative impact, negative problem due to poor quality of the product for personal indoor and outdoor mobility and transportation.
-4 - Complete barrier: 96–100% negative impact, negative problem and cannot be prevented, as there is no suitable product for personal indoor and outdoor mobility and transportation, or it is unavailable.
0 - No or very low facilitator or facilitator: 0–4%.
+1 - Mild facilitator: 5–24% positive effect of product for personal indoor and outdoor mobility and transportation, but product access is variable, its effect is inconsistent, or the product does not facilitate all functions.
+2 - Moderate facilitator: 25–49% positive effect of personal indoor and outdoor mobility and transportation; product is available, but its effect is not fully consistent, and it does not facilitate all functions.
+3 - Substantial facilitator: 50–95% positive effect of product for personal indoor and outdoor mobility and transportation; product is available, its effect is consistent, the quality is good, and it helps facilitate most functions.
+4 - Complete facilitator: 96–100% positive effect of product for personal indoor and outdoor mobility and transportation; product is available, its effect is consistent, the quality is high, and it helps facilitate all functions.

e125 Products and technology for communication
Equipment, products and technologies used by people in activities of sending and receiving information, including those adapted or specially designed, in, on or near the person using them.
Inclusions: General and assistive products and technology for communication.

0 - No or very low barrier: 0–4%.

-1 - Mild barrier: 5–24% negative impact, but always avoidable, must purchase a product for communication.

-2 – Moderate barrier: 25–49% negative impact, but can be prevented by prescribing or referring to products for communication.

-3 – Severe barrier: 50–95% negative impact due to poor quality of the product meant for communication.

-4 - Complete barrier: 96–100% negative impact and cannot be prevented, as there is no suitable product for communication or it is unavailable.

0 - No or very low facilitator: 0–4%.

+1 - Mild facilitator: 5–24% positive effect of product for communication, but product access is variable, the effect is inconsistent or it does not facilitate all functions.

+2 - Moderate facilitator: 25–49% positive effect of product for communication; product is available, but the effect is not fully consistent and it does not facilitate all functions.

+3 - Substantial facilitator: 50–95% positive effect of product for communication; product is available, the effect is consistent, the quality is good, and it helps facilitate most functions.

+4 - Complete facilitator: 96–100% positive effect of product for communication; product is available, the effect is consistent, the quality is high, and it helps facilitate all functions.

e135 Products and technology for employment

Equipment, products and technology used for employment to facilitate work activities.

Inclusion: general and assistive products and technology for employment

0 - No or very low barrier: 0–4%.

-1 - Mild barrier: 5–24% negative impact, 5–24% problem, but always avoidable, must purchase a product for employment.

-2 – Moderate barrier: 25–49% negative impact, 25–49% problem, but can be prevented by prescribing or referring to product for employment.

-3 – Severe barrier: 50–95% negative impact, 50–95% negative problem due to poor quality of the product meant for employment.

-4 - Complete barrier: 96–100% negative impact, 96–100% negative problem and cannot be prevented as there is no suitable product for employment available.

0 - No or very low facilitator: 0–4%.

+1 - Mild facilitator: 5–24% positive effect of product for employment, but product access is variable and its effect is inconsistent or the product does not facilitate all activities.

+2 - Moderate facilitator: 25–49% positive effect of product for employment; product is available, but the effect is not fully consistent and it does not facilitate all activities.
+3 - Substantial facilitator: 50–95% positive effect of product for employment; product is available, the effect is consistent, the quality is good, and it helps facilitate most activities.
+4 - Complete facilitator: 96–100% positive effect of product for employment; product is available, the effect is consistent, the quality is high, and it helps facilitate all activities.

e150 Design, construction and building products and technology of buildings for public use
Products and technology that constitute an individual's indoor and outdoor human-made environment that is planned, designed and constructed for public use, including those adapted or specially designed.
Inclusions: Design, construction and building products and technology of entrances and exits, facilities and routing.

0 - No or very low barrier: 0–4%.
-1 - Mild barrier: 5–24% negative impact; accessibility features are installed at a few public areas, but it is possible to avoid a hindrance.
-2 - Moderate barrier: 25–49% negative impact; accessibility features are installed only at individual areas but can be addressed.
-3 - Severe barrier: 50–95% negative impact and cannot be prevented; accessibility features are rare in public areas and are of poor quality.
-4 - Complete barrier: 96–100% negative impact, the problem cannot be prevented, and no accessibility features are available in public areas.
0 - No or very low facilitator: 0–4%.
+1 - Mild facilitator: 5–24% positive effect. Accessibility features are installed sparsely, varying quality, and adapted only for a narrow group of persons with disability.
+2 - Moderate facilitator: 25–49% positive effect. Accessibility features are installed at some public buildings, are of quality, and are adapted only for a narrow group of persons with disability.
+3 - Substantial facilitator: 50–95% positive effect. Accessibility features are installed in major public areas, are of good quality, and partially relieve or neutralize a high variety of disabilities.
+4 - Complete facilitator: 96–100% positive effect. Accessibility features are widely installed in public areas, are of high quality, and fully relieve or neutralize a high variety of disabilities.

e155 Design, construction and building products and technology of buildings for private use

Products and technology that constitute an individual's indoor and outdoor human-made environment that is planned, designed and constructed for private use, including those adapted or specially designed.

Inclusions: Design, construction and building of products and the technology of entrances and exits, facilities and routing.

e1550 Design, construction and building products and technology for entering and exiting of buildings for private use

Products and technology of entry and exit from the human made environment that is planned, designed and constructed for private use, such as entries and exits to private homes, portable and stationary ramps, power-assisted doors, lever door handles and level door thresholds.

0 - No or very low barrier: 0–4%.

-1 - Mild barrier: 5–24% negative impact; accessibility features are installed at a few private areas, but it is possible to avoid a hindrance.

-2 - Moderate barrier: 25–49% negative impact; accessibility features are installed only at individual areas but can be addressed.

-3 - Severe barrier: 50–95% negative impact and cannot be prevented; accessibility features are rare in private areas and are of poor quality.

-4 - Complete barrier: 96–100% negative impact, the problem cannot be prevented, and no accessibility features are available in private areas.

0 - No or very low facilitator: 0–4%.

+1 - Mild facilitator: 5–24% positive effect. Accessibility features are installed sparsely, varying quality, and adapted only for a narrow group of persons with disability.

+2 - Moderate facilitator: 25–49% positive effect. Accessibility features are installed at some private buildings, are of quality, and are adapted only for a narrow group of persons with disability.

+3 - Substantial facilitator: 50–95% positive effect. Accessibility features are installed in major private areas, are of good quality, and partially relieve or neutralize a high variety of disabilities.

+4 - Complete facilitator: 96–100% positive effect. Accessibility features are widely installed in private areas, are of high quality, and fully relieve or neutralize a high variety of disabilities.

e165 Assets

Products or objects of economic exchange such as money, goods, property and other valuables that an individual owns or of which he or she has rights of use.

Inclusions: Tangible and intangible products and goods and financial assets.

e1650 Financial assets

Products, such as money and other financial instruments, serve as a medium of exchange for labour, capital goods and services.

0 - No or very low barrier: 0–4%.
-1 - Mild barrier: 5–24% negative impact. Accessibility features are rarely applied on physical and digital money forms, but it is possible to avoid hindrance.
-2 - Moderate barrier: 25–49% negative impact. Accessibility features are rarely applied on physical and digital money forms, but it is possible to avoid hindrance with the assistance of other people.
-3 - Severe barrier: 50–95% negative impact; accessibility features are low quality and the assistance of other people is not available or has a negative effect.
-4 - Complete barrier: 96–100% negative impact; no accessibility features or assistance is available.
0 - No or very low facilitator: 0–4%.
+1 - Mild facilitator: 5–24% positive effect. Accessibility features are only applied to some physical and digital money forms. Only basic financial asset transaction types are adapted.
+2 - Moderate facilitator: 25–49% positive effect. Accessibility features are mainly applied in physical money forms and rarely in digital forms. A few financial asset transaction types are adapted to relieve or neutralize disability, and financial services are rarely adapted for persons with disability.
+3 - Substantial facilitator: 50–95% positive effect. Accessibility features are applied widely in physical and digital money forms; only main financial asset transactions are fully adapted to relieve or neutralize disability, and financial advice and assistance are rarely adapted for persons with disability.
+4 - Complete facilitator: 96–100% positive effect. Accessibility features are present in physical and digital money forms, a wide variety of financial asset transactions are fully adapted to fully relieve or neutralize disability, and financial advice and assistance are adapted for persons with disability.

e2250 Temperature
Degree of heat or cold, such as high and low temperature, normal or extreme temperature.

0 - No or very low barrier: approximately 0–4%.
-1 - Mild barrier: 5–24% negative impact. Little ability to adjust ambient temperature, but it is possible to avoid hindrance.
-2 - Moderate barrier: 25–49% negative impact. Low ability to adjust ambient temperature; clothing and means are low quality and are not always available.
-3 - Severe barrier: 50–95% negative impact. Low ability to adjust ambient temperature; clothing and means are poor quality and sparsely available.

-4 - Complete barrier: 96–100% negative impact. No ability to adjust ambient temperature, no available clothing or other means.
0 - No or very low facilitator: approximately 0–4%.
+1 - Mild facilitator: 5–24% positive effect. There is a narrow ability to adjust ambient temperature and access to clothing for mild temperature control.
+2 - Moderate facilitator: 25–49% positive effect. There is availability and means to adjust ambient temperature and access to clothing for mild temperature control.
+3 - Substantial facilitator: 50–95% positive effect. There is availability and means to adjust ambient temperature or to insulate using clothing or other means; however, it is not always available for extreme temperatures.
+4 – Complete facilitator: 96–100% positive effect. There is always availability and means to adjust ambient temperature or to insulate using clothing or other means on a wide amplitude of temperatures.

e2251 Humidity
Level of moisture in the air, such as high or low humidity.

0 - No or very low barrier: approximately 0–4%.
-1 - Mild barrier: 5–24% negative impact. Little ability to adjust ambient humidity, but it is possible to avoid hindrance.
-2 - Moderate barrier: 25–49% negative impact. Low ability to adjust ambient humidity; clothing and means are low quality and are not always available.
-3 - Severe barrier: 50–95% negative impact. Low ability to adjust ambient humidity; clothing and means are poor quality and are sparsely available.
-4 - Complete barrier: 96–100% negative impact. No ability to adjust ambient humidity and no available clothing or other means.
0 - No or very low facilitator: approximately 0–4%.
+1 - Mild facilitator: 5–24% positive effect. There is a narrow ability to adjust ambient humidity and access to clothing for mild humidity control.
+2 - Moderate facilitator: 25–49% positive effect. There is availability and means to adjust ambient humidity and access to clothing for mild humidity control.
+3 - Substantial facilitator: 50–95% positive effect. There is availability and means to adjust humidity or to use clothing or other means; however, it is not always available for extreme humidity.
+4 – Complete facilitator: 96–100% positive effect. There is always availability and means to adjust ambient humidity or to use clothing or other means on a wide amplitude of humidity.

e2253 Precipitation

Falling of moisture, such as rain, dew, snow, sleet and hail.

0 - No or very low barrier: approximately 0–4%.

-1 - Mild barrier: 5–24% negative impact. Little ability to adjust to precipitation, but it is possible to avoid hindrance.

-2 - Moderate barrier: 25–49% negative impact. Low ability to adjust to precipitation; clothing and means are low quality and are not always available.

-3 - Severe barrier: 50–95% negative impact. Low ability to adjust to precipitation; clothing and means are poor quality and are sparsely available.

-4 - Complete barrier: 96–100% negative impact. No ability to adjust to precipitation; no available clothing or other means.

0 - No or very low facilitator: approximately 0–4%.

+1 - Mild facilitator: 5–24% positive effect. There is a minimal ability to adjust to precipitation and access clothing designed for basic precipitation (rain).

+2 - Moderate facilitator: 25–49% positive effect. There is little availability of clothing and other means designed for basic precipitation (snow and rain) that are of good quality.

+3 - Substantial facilitator: 50–95% positive effect. There is good availability of clothing and other means designed for precipitation; however, they are not always available for extreme precipitation.

+4 – Complete facilitator: 96–100% positive effect. There is always availability of clothing and other means to adjust to all types of precipitation.

e310 Immediate family.

Individuals related by birth, marriage, or other relationships recognized by the culture as immediate family, such as spouses, partners, parents, siblings, children, foster parents, adoptive parents, and grandparents. Evaluation of physical and emotional support within the family and relationship with them at home.

0 - No or very low barrier: 0–4%.

–1 – Mild barrier: 5–24% negative impact, immediate family members are inconsistently an emotional and/or physical burden, but it is possible to avoid them or mitigate the effect.

–2 – Moderate barrier: 25–49% negative impact, immediate family members are an occasional emotional and/or physical burden, and it is not always possible to avoid them or mitigate the effect.

–3 – Severe barrier: 50–95% negative impact, immediate family members are a constant emotional and/or physical burden, and it is not always possible to avoid them or mitigate the effect.
–4 – Complete barrier: 96–100% hindrance, immediate family members are a constant emotional and/or physical burden, and it is not possible to avoid them or mitigate the effect.
0 - No or very low facilitator: 0–4%.
+1 – Mild facilitator: 5–24% positive effect; immediate family members provide either physical or emotional support, but it is inconsistent, not always available and/or help is low quality.
+2 – Moderate facilitator: 25–49% positive effect; immediate family members provide practical physical and emotional support and are inconsistently helping, but help is always of good quality.
+3 – Substantial facilitator: 50–95% positive effect; immediate family members provide practical physical and emotional support, are always helping by promoting functioning and help is always of good quality.
+4 – Complete facilitator: 96–100% positive effect; immediate family members always provide practical physical and emotional support by promoting functioning and decreasing disability, and help is of high quality.

e315 Extended family

Individuals related through family or marriage or other relationships recognized by the culture as extended family, such as aunts, uncles, nephews and nieces.
Exclusion: Immediate family (e310)

0 - No or very low barrier: 0–4%.
–1 – Mild barrier: 5–24% negative impact, extended family members are inconsistently an emotional and/or physical burden, but it is possible to avoid them or mitigate the effect.
–2 – Moderate barrier: 25–49% negative impact, extended family members are an occasional emotional and/or physical burden, and it is not always possible to avoid them or mitigate the effect.
–3 – Severe barrier: 50–95% negative impact, extended family members are a constant emotional and/or physical burden, and it is not always possible to avoid them or mitigate the effect.
–4 – Complete barrier: 96–100% hindrance, extended family members are a constant emotional and/or physical burden, and it is not possible to avoid them or mitigate the effect.
0 - No or very low facilitator: 0–4%.
+1 – Mild facilitator: 5–24% positive effect; extended family members provide either physical or emotional support, but it is inconsistent, not always available and/or help is low quality.
+2 – Moderate facilitator: 25–49% positive effect; extended family members provide practical physical and emotional support and are inconsistently helping, but help is always of good quality.

+3 – Substantial facilitator: 50–95% positive effect; extended family members provide practical physical and emotional support, are always helping by promoting functioning, and help is always of good quality.
+4 – Complete facilitator: 96–100% positive effect; extended family members always provide practical physical and emotional support by promoting functioning and decreasing disability, and help is of high quality.

e320 Friends
Individuals who are close and ongoing participants in relationships characterized by trust and mutual support.

0 - No or very low barrier: 0–4%.
–1 – Mild barrier: 5–24% negative impact, friends are inconsistently an emotional and/or physical burden, but it is possible to avoid them or mitigate the effect.
–2 – Moderate barrier: 25–49% negative impact, friends are an occasional emotional and/or physical burden, and it is not always possible to avoid them or mitigate the effect.
–3 – Severe barrier: 50–95% negative impact, friends are a constant emotional and/or physical burden, and it is not always possible to avoid them or mitigate the effect.
–4 – Complete barrier: 96–100% hindrance, friends are a constant emotional and/or physical burden, and it is not possible to avoid them or mitigate the effect.
0 - No or very low facilitator: 0–4%.
+1 – Mild facilitator: 5–24% positive effect; friends provide either physical or emotional support, but it is inconsistent, not always available and/or help is low quality.
+2 – Moderate facilitator: 25–49% positive effect; friends provide practical physical and emotional support and are inconsistently helping, but help is always of good quality.
+3 – Substantial facilitator: 50–95% positive effect; friends provide practical physical and emotional support, are always helping by promoting functioning and help is always of good quality.
+4 – Complete facilitator: 96–100% positive effect; friends always provide practical physical and emotional support by promoting functioning and decreasing disability, and help is of high quality.

e325 Acquaintances, peers, colleagues, neighbours and community members
Individuals who are familiar to each other as acquaintances, peers, colleagues, neighbours, and community members in situations of work, school, recreation, or other aspects of life, and who share demographic features such as age, gender, religious creed or ethnicity or pursue common interests.

0 - No or very low barrier: approximately 0–4%.

–1 – Mild barrier: 5–24% negative impact, acquaintances, peers, colleagues, neighbours are inconsistently an emotional and/or physical burden, but it is possible to avoid them or mitigate the effect.
–2 – Moderate barrier: 25–49% negative impact, acquaintances, peers, colleagues, neighbours are an occasional emotional and/or physical burden, and it is not always possible to avoid them or mitigate the effect.
–3 – Severe barrier: 50–95% negative impact, acquaintances, peers, colleagues, neighbours are a constant emotional and/or physical burden, and it is not always possible to avoid them or mitigate the effect.
–4 – Complete barrier: 96–100% hindrance, acquaintances, peers, colleagues, neighbours are a constant emotional and/or physical burden, and it is not possible to avoid them or mitigate the effect.
0 - No or very low facilitator: approximately 0–4%.
+1 – Mild facilitator: 5–24% positive effect; acquaintances, peers, colleagues, neighbours provide either physical or emotional support, but it is inconsistent, not always available and/or help is low quality.
+2 – Moderate facilitator: 25–49% positive effect; acquaintances, peers, colleagues, neighbours provide practical physical and emotional support and are inconsistently helping, but help is always of good quality.
+3 – Substantial facilitator: 50–95% positive effect; acquaintances, peers, colleagues, neighbours provide practical physical and emotional support, are always helping by promoting functioning and help is always of good quality.
+4 – Complete facilitator: 96–100% positive effect; acquaintances, peers, colleagues, neighbours always provide practical physical and emotional support by promoting functioning and decreasing disability and help is of high quality.

e330 People in positions of authority
Individuals who have decision-making responsibilities for others and who have socially defined influence or power based on their social, economic, cultural or religious roles in society, such as teachers, employers, supervisors, religious leaders, substitute decision-makers, guardians or trustees.

0 - No or very low barrier: 0–4%.
–1 – Mild barrier: 5–24% negative impact, people in positions of authority are inconsistently an emotional and/or physical burden, but it is possible to avoid them or mitigate the effect.
–2 – Moderate barrier: 25–49% negative impact, people in positions of authority are an occasional emotional and/or physical burden, and it is not always possible to avoid them or mitigate the effect.
–3 – Severe barrier: 50–95% negative impact, people in positions of authority are a constant emotional and/or physical burden, and it is not always possible to avoid them or mitigate the effect.

–4 – Complete barrier: 96–100% hindrance, people in positions of authority are a constant emotional and/or physical burden, and it is not possible to avoid them or mitigate the effect.
0 - No or very low facilitator: 0–4%.
+1 – Mild facilitator: 5–24% positive effect; people in positions of authority provide either physical or emotional support, but it is inconsistent, not always available and/or help is low quality.
+2 – Moderate facilitator: 25–49% positive effect; people in positions of authority provide practical physical and emotional support and are inconsistently helping, but help is always of good quality.
+3 – Substantial facilitator: 50–95% positive effect; people in positions of authority provide practical physical and emotional support, are always helping by promoting functioning, and help is always of good quality.
+4 – Complete facilitator: 96–100% positive effect; people in positions of authority always provide practical physical and emotional support by promoting functioning and decreasing disability, and help is of high quality.
has impairments in functioning and disability.

e340 Personal care providers and personal assistants
Individuals who provide services as required to support individuals in their daily activities and maintenance of performance at work, education or other life situations, provided either through public or private funds, or else on a voluntary basis, such as providers of support for home-making and maintenance, personal assistants, transport assistants, paid help, nannies and others who function as primary caregivers.

0 - No or very low barrier: 0–4%.
–1 – Mild barrier: 5–24% negative impact, personal care providers and personal assistants are inconsistently an emotional and/or physical burden, but it is possible to avoid them or mitigate the effect.
–2 – Moderate barrier: 25–49% negative impact, personal care providers and personal assistants are an occasional emotional and/or physical burden and it is not always possible to avoid them or mitigate the effect.
–3 – Severe barrier: 50–95% negative impact, personal care providers and personal assistants are a constant emotional and/or physical burden, and it is not always possible to avoid them or mitigate the effect.
–4 – Complete barrier: 96–100% hindrance, personal care providers and personal assistants are a constant emotional and/or physical burden, and it is not possible to avoid them or mitigate the effect.
0 - No or very low facilitator: 0–4%.
+1 – Mild facilitator: 5–24% positive effect; personal care providers and personal assistants provide either physical or emotional support, but it is inconsistent, not always available and/or help is low quality.

+2 – Moderate facilitator: 25–49% positive effect; personal care providers and personal assistants provide practical physical and emotional support and are inconsistently helping, but help is always of good quality.
+3 – Substantial facilitator: 50–95% positive effect; personal care providers and personal assistants provide practical physical and emotional support, are always helping by promoting functioning, and help is always of good quality.
+4 – Complete facilitator: 96–100% positive effect; personal care providers and personal assistants always provide practical physical and emotional support by promoting functioning and decreasing disability, and help is of high quality.
and disability.

e355 Health professionals.
All service providers working within the context of the health system, such as doctors, nurses, physiotherapists, occupational therapists, speech therapists, audiologists, orthotist-prosthetists, and medical social workers. Evaluation of physical and emotional support within health professionals and relationship with them.

0 - No or very low barrier: 0–4%.
–1 – Mild barrier: 5–24% negative impact, health professionals are inconsistently an emotional and/or physical burden, but it is possible to avoid them or mitigate the effect.
–2 – Moderate barrier: 25–49% negative impact, health professionals are an occasional emotional and/or physical burden, and it is not always possible to avoid them or mitigate the effect.
–3 – Severe barrier: 50–95% negative impact, health professionals are a constant emotional and/or physical burden and it is not always possible to avoid them or mitigate the effect.
–4 – Complete barrier: 96–100% hindrance, health professionals are a constant emotional and/or physical burden, and it is not possible to avoid them or mitigate the effect.
0 - No or very low facilitator: 0–4%.
+1 – Mild facilitator: 5–24% positive effect; health professionals provide either physical or emotional support, but it is inconsistent, not always available and/or help is low quality.
+2 – Moderate facilitator: 25–49% positive effect; health professionals provide practical physical and emotional support and are inconsistently helping, but help is always of good quality.
+3 – Substantial facilitator: 50–95% positive effect; health professionals provide practical physical and emotional support, are always helping by promoting functioning, and help is always of good quality.
+4 – Complete facilitator: 96–100% positive effect; health professionals always provide practical physical and emotional support by promoting functioning and decreasing disability, and help is of high quality.

e360 Other professionals

All service providers working outside the health system, including *social workers*, lawyers, teachers, architects and designers.

0 - No or very low barrier: 0–4%.

–1 – Mild barrier: 5–24% negative impact, social workers are inconsistently an emotional and/or physical burden, but it is possible to avoid them or mitigate the effect.

–2 – Moderate barrier: 25–49% negative impact, social workers are an occasional emotional and/or physical burden, and it is not always possible to avoid them or mitigate the effect.

–3 – Severe barrier: 50–95% negative impact, social workers are a constant emotional and/or physical burden, and it is not always possible to avoid them or mitigate the effect.

–4 – Complete barrier: 96–100% hindrance, social workers are a constant emotional and/or physical burden, and it is not possible to avoid them or mitigate the effect.

0 - No or very low facilitator: 0–4%.

+1 – Mild facilitator: 5–24% positive effect; social workers provide either physical or emotional support, but it is inconsistent, not always available and/or help is low quality.

+2 – Moderate facilitator: 25–49% positive effect; social workers provide practical physical and emotional support and are inconsistently helping, but help is always of good quality.

+3 – Substantial facilitator: 50–95% positive effect; social workers provide practical physical and emotional support, are always helping by promoting functioning, and help is always of good quality.

+4 – Complete facilitator: 96–100% positive effect; social workers always provide practical physical and emotional support by promoting functioning and decreasing disability, and help is of high quality.

e410 Individual attitudes of immediate family members.

General or specific opinions and beliefs of immediate family members about the person or about other matters (e.g., social, political, and economic issues) that influence individual behaviour and actions.

0 - No or very low barrier: 0–4%.

–1 – Mild barrier: 5–24% negative impact, there are negative individual attitudes of immediate family members, but they are almost unnoticed, or they do not affect the person`s behaviour or social life.

–2 – Moderate barrier: 25–49% negative impact, there are negative individual attitudes of immediate family members. They are almost always present, and they do not affect the person’s behaviour or social life, as the person mitigates them and thus does not suffer from negative attitudes.

–3 – Severe barrier: 50–95% negative impact, there are negative individual attitudes of immediate family members. They are always present, and the person can rarely avoid them. They affect a person's behaviour and social life, and the person suffers from negative attitudes.
–4 – Complete barrier: 96–100% negative impact, there are negative individual attitudes of immediate family members. They are always present and discriminating, there is no possibility to avoid them, and they affect the person's behaviour and social life, restricting equal opportunities.
0 - No or very low facilitator: 0–4%.
+1 – Mild facilitator: 5–24% support and positive attitudes, respect for the person, positive motivation.
+2 – Moderate facilitator: 25–49% support and positive attitudes that positively affect the person´s behaviour.
+3 – Substantial facilitator: 50–95% support and positive attitudes that positively affect the person´s behaviour and social life.
+4 – Complete facilitator: 96–100% support and positive and honorific attitudes in all relationships.

e415 Individual attitudes of extended family members
General or specific opinions and beliefs of extended family members about the person or about other matters (e.g., social, political and economic issues) that influence individual behaviour and actions.

0 - No or very low barrier: 0–4%.
–1 – Mild barrier: 5–24% negative impact, there are negative individual attitudes of extended family members, but they are almost unnoticed, or they do not affect the person`s behaviour or social life.
–2 – Moderate barrier: 25–49% negative impact, there are negative individual attitudes of extended family members. They are almost always present, but they do not affect the person's behaviour or social life as the person mitigates them and thus does not suffer from negative attitudes.
–3 – Severe barrier: 50–95% negative impact, there are negative individual attitudes of extended family members. They are always present, and the person can rarely avoid them. They affect the person's behaviour and social life, and the person suffers from negative attitudes.
–4 – Complete barrier: 96–100% negative impact, there are negative individual attitudes of extended family members. They are always present and discriminating, there is no possibility to avoid them, and they affect the person's behaviour and social life, restricting equal opportunities.
0 - No or very low facilitator: 0–4%.
+1 – Mild facilitator: 5–24% support and positive attitudes, respect for the person, positive motivation.
+2 – Moderate facilitator: 25–49% support and positive attitudes that positively affect the person´s behaviour.

+3 – Substantial facilitator: 50–95% support and positive attitudes that positively affect the person´s behaviour and social life.
+4 – Complete facilitator: 96–100% support and positive and honorific attitudes in all relationships.
+4 – Complete facilitator: 96–100% support and positive attitudes in all relationships.

e420 Individual attitudes of friends

General or specific opinions and beliefs of friends about the person or about other matters (e.g., social, political and economic issues) that influence individual behaviour and actions.

0 - No or very low barrier: 0–4%.
–1 – Mild barrier: 5–24% negative impact, there are negative individual attitudes of friends, but they are almost unnoticed, or they do not affect the person`s behaviour or social life.
–2 – Moderate barrier: 25–49% negative impact, there are negative individual attitudes of friends. They are almost always present, but they do not affect the person's behaviour or social life as the person mitigates them and thus does not suffer from negative attitudes.
–3 – Severe barrier: 50–95% negative impact, there are negative individual attitudes of friends. They are always present, and the person can rarely avoid them. They affect the person's behaviour and social life, and the person suffers from negative attitudes.
–4 – Complete barrier: 96–100% negative impact, there are negative individual attitudes of friends. They are always present and discriminating, there is no possibility to avoid them, and they affect the person's behaviour and social life, restricting equal opportunities.
0 - No or very low facilitator: 0–4%.
+1 – Mild facilitator: 5–24% support and positive attitudes, respect for the person, positive motivation.
+2 – Moderate facilitator: 25–49% support and positive attitudes that positively affect the person´s behaviour.
+3 – Substantial facilitator: 50–95% support and positive attitudes that positively affect the person´s behaviour and social life.
+4 – Complete facilitator: 96–100% support and positive and honorific attitudes in all relationships.

e425 Individual attitudes of acquaintances, peers, colleagues, neighbours and community members

General or specific opinions and beliefs of acquaintances, peers, colleagues, neighbours and community members about the person or about other matters (e.g. social, political and economic issues) that influence individual behaviour and actions.

0 - No or very low barrier: approximately 0–4%.
–1 – Mild barrier: 5–24% negative impact, there are negative individual attitudes of acquaintances, peers, colleagues, neighbours, but they are almost unnoticed, or they do not affect the person`s behaviour or social life.
–2 – Moderate barrier: 25–49% negative impact, there are negative individual attitudes of acquaintances, peers, colleagues, neighbours. They are almost always present, but they do not affect the person`s behaviour or social life as the person mitigates them and thus does not suffer from negative attitudes.
–3 – Severe barrier: 50–95% negative impact, there are negative individual attitudes of acquaintances, peers, colleagues, neighbours. They are always present, and the person can rarely avoid them. They affect the person`s behaviour and social life, and the person suffers from negative attitudes.
–4 – Complete barrier: 96–100% negative impact, there are negative individual attitudes of acquaintances, peers, colleagues, neighbours. They are always present and discriminating, there is no possibility to avoid them, and they affect the person`s behaviour and social life, restricting equal opportunities.
0 - No or very low facilitator: approximately 0–4%.
+1 – Mild facilitator: 5–24% support and positive attitudes, respect to the person, positive motivation.
+2 – Moderate facilitator: 25–49% support and positive attitudes that positively affect the person´s behaviour.
+3 – Substantial facilitator: 50–95% support and positive attitudes that positively affect the person´s behaviour and social life.
+4 – Complete facilitator: 96–100% support and positive and honorific attitudes in all relationships.

e430 Individual attitudes of people in positions of authority
General or specific opinions and beliefs of people in positions of authority about the person or about other matters (e.g., social, political and economic issues) that influence individual behaviour and actions.

0 - No or very low barrier: 0–4%.
–1 – Mild barrier: 5–24% negative impact, there are negative individual attitudes of people in positions of authority, but they are almost unnoticed, or they do not affect the person`s behaviour or social life.
–2 – Moderate barrier: 25–49% negative impact, there are negative individual attitudes of people in positions of authority. They are almost always present but they do not affect the person's behaviour or social life as the person mitigates them and thus does not suffer from negative attitudes.
–3 – Severe barrier: 50–95% negative impact, there are negative individual attitudes of people in positions of authority. They are always present, and the person can rarely avoid them. They affect the person's behaviour and social life, and the person suffers from negative attitudes.

–4 – Complete barrier: 96–100% negative impact, there are negative individual attitudes of people in positions of authority. They are always present and discriminating, there is no possibility to avoid them, and they affect the person's behaviour and social life, restricting equal opportunities.
0 - No or very low facilitator: 0–4%.
+1 – Mild facilitator: 5–24% support and positive attitudes, respect for the person, positive motivation.
+2 – Moderate facilitator: 25–49% support and positive attitudes that positively affect the person´s behaviour.
+3 – Substantial facilitator: 50–95% support and positive attitudes that positively affect the person´s behaviour and social life.
+4 – Complete facilitator: 96–100% support and positive and honorific attitudes in all relationships.

e440 Individual attitudes of personal care providers and personal assistants
General or specific opinions and beliefs of personal care providers and personal assistants about the person or about other matters (e.g., social, political and economic issues) that influence individual behaviour and actions.

0 - No or very low barrier: 0–4%.
–1 – Mild barrier: 5–24% negative impact, there are negative individual attitudes of personal care providers and personal assistants, but they are almost unnoticed, or they do not affect the person`s behaviour or social life.
–2 – Moderate barrier: 25–49% negative impact, there are negative individual attitudes of personal care providers and personal assistants. They are almost always present, but they do not affect the person's behaviour or social life as the person mitigates them and thus does not suffer from negative attitudes.
–3 – Severe barrier: 50–95% negative impact, there are negative individual attitudes of personal care providers and personal assistants. They are always present, and the person can rarely avoid them. They affect the person's behaviour and social life, and the person suffers from negative attitudes.
–4 – Complete barrier: 96–100% negative impact, there are negative individual attitudes of personal care providers and personal assistants. They are always present and discriminating, there is no possibility to avoid them, and they affect the person's behaviour and social life, restricting equal opportunities.
0 - No or very low facilitator: 0–4%.
+1 – Mild facilitator: 5–24% support and positive attitudes, respect for the person, positive motivation.
+2 – Moderate facilitator: 25–49% support and positive attitudes that positively affect the person´s behaviour.

+3 – Substantial facilitator: 50–95% support and positive attitudes that positively affect the person´s behaviour and social life.
+4 – Complete facilitator: 96–100% support and positive and honorific attitudes in all relationships.

e450 Individual attitudes of health professionals. General or specific opinions and beliefs of health professionals about the person or about other matters (e.g., social, political, and economic issues) that influence individual behaviour and actions.

0 - No or very low barrier: 0–4%.
–1 – Mild barrier: 5–24% negative impact, there are negative individual attitudes of health professionals, but they are almost unnoticed, or they do not affect the person`s behaviour or social life.
–2 – Moderate barrier: 25–49% negative impact, there are negative individual attitudes of health professionals. They are almost always present, but they do not affect the person's behaviour or social life as the person mitigates them and thus does not suffer from negative attitudes.
–3 – Severe barrier: 50–95% negative impact, there are negative individual attitudes of health professionals. They are always present, and the person can rarely avoid them. They affect a person's behaviour and social life, and the person suffers from negative attitudes.
–4 – Complete barrier: 96–100% negative impact, there are negative individual attitudes of health professionals. They are always present and discriminating, there is no possibility to avoid them, and they affect the person's behaviour and social life, restricting equal opportunities.
0 - No or very low facilitator: 0–4%.
+1 – Mild facilitator: 5–24% support and positive attitudes, respect for the person, positive motivation.
+2 – Moderate facilitator: 25–49% support and positive attitudes that positively affect the person´s behaviour.
+3 – Substantial facilitator: 50–95% support and positive attitudes that positively affect the person´s behaviour and social life.
+4 – Complete facilitator: 96–100% support and positive and honorific attitudes in all relationships.

e460 Societal attitudes
General or specific opinions and beliefs generally held by people of a culture, society, subcultural or other social group about other individuals or about other social, political and economic issues that influence group or individual behaviour and actions.

0 - No or very low barrier: 0–4%.

–1 – Mild barrier: 5–24% negative impact, there are negative societal attitudes, but they are almost unnoticed, or they do not affect the person`s behaviour or social life.
–2 – Moderate barrier: 25–49% negative impact, there are negative societal attitudes. They are almost always present, but they do not affect the person's behaviour or social life as the person mitigates them and thus does not suffer from negative attitudes.
–3 – Severe barrier: 50–95% negative impact, there are negative societal attitudes. They are always present, and the person can rarely avoid them. They affect the person's behaviour and social life, and the person suffers from negative attitudes.
–4 – Complete barrier: 96–100% negative impact, there are negative societal attitudes. They are always present and discriminating, there is no possibility to avoid them, and they affect the person's behaviour and social life, restricting equal opportunities.
0 - No or very low facilitator: 0–4%.
+1 – Mild facilitator: 5–24% support and positive societal attitudes, respect for the person, positive motivation.
+2 – Moderate facilitator: 25–49% support and positive societal attitudes that positively affect the person´s behaviour.
+3 – Substantial facilitator: 50–95% support and positive societal attitudes that positively affect the person´s behaviour and social life.
+4 – Complete facilitator: 96–100% support and societal positive and honorific attitudes in all relationships.

e515 Architecture and construction services, systems and policies

Services, systems and policies for the design and construction of buildings, public and private.

0 - No or very low barrier: 0–4%.
-1 - Mild barrier: 5–24% negative effect; architecture and construction services are inconsistently available, but services are of inconsistent quality.
-2 - Moderate barrier: 25–49% negative effect; architecture and construction services are sparsely available, and services are of inconsistent quality.
-3 - Severe barrier: 50–95% negative effect; architecture and construction services are sparsely available, and services are of low quality.
-4 - Complete barrier: 96–100% negative effect; architecture and construction services are not available.
0 - No or very low facilitator: 0–4%.
+1 - Mild facilitator: 5–24% positive effect; architecture and construction services are available, access is variable, but services do not adapt well to the person's needs.

+2 - Moderate facilitator: 25–49% positive effect; architecture and construction services are available and widely accessible, but services only partly satisfy the person’s needs
+3 - Substantial facilitator: 50–95% positive effect architecture and construction services are available, widely accessible, of good quality, and almost fully satisfy the person’s needs.
+4 - Complete facilitator: 96–100% positive effect; architecture and construction services are available, widely accessible, of high quality, and fully satisfy the person’s needs.

e525 Housing services, systems and policies
Services, systems and policies for the provision of shelters, dwellings or lodging for people.

e5250 Housing services
Services and programmes aimed at locating, providing and maintaining houses or shelters for persons to live in, such as estate agencies, housing organizations, and shelters for homeless people, including those who provide these services.

0 - No or very low barrier: 0–4%.
-1 - Mild barrier: 5–24% negative effect; housing services are inconsistently available, but services are of inconsistent quality.
-2 - Moderate barrier: 25–49% negative effect; housing services are sparsely available, and services are of inconsistent quality.
-3 - Severe barrier: 50–95% negative effect; housing services are sparsely available, and services are of low quality.
-4 - Complete barrier: 96–100% negative effect; housing services are not available.
0 - No or very low facilitator: approximately 0–4%.
+1 - Mild facilitator: 5–24% positive effect; housing services are available, access is variable, but services do not adapt well to the needs of the person.
+2 - Moderate facilitator: 25–49% positive effect; housing services are available and widely accessible, but services only partly satisfy the person's needs,
+3 - Substantial facilitator: 50–95% positive effect; housing services are available, widely accessible, of good quality, and almost fully satisfy the person's needs.
+4 - Complete facilitator: 96–100% positive effect; housing services are available, widely accessible, of high quality, and fully satisfy the person's needs.

e540 Transportation services, systems and policies

Services, systems and policies for enabling people or goods to move or be moved from one location to another.

e5400 Transportation services

Services and programmes aimed at moving persons or goods by road, paths, rail, air or water, by public or private transport, including those who provide these services.

0 - No or very low barrier: 0–4%.

-1 - Mild barrier: 5–24% negative effect; transportation services are inconsistently available, but services are of inconsistent quality.

-2 - Moderate barrier: 25–49% negative effect; transportation services are sparsely available, and services are of inconsistent quality.

-3 - Severe barrier: 50–95% negative effect; transportation services are sparsely available and services are of low quality.

-4 - Complete barrier: 96–100% negative effect; transportation services are not available.

0 - No or very low facilitator: 0–4%.

+1 - Mild facilitator: 5–24% positive effect; transportation services are available, access is variable, but services do not adapt well to the person’s needs.

+2 - Moderate facilitator: 25–49% positive effect; transportation services are available, widely accessible, but services only partly satisfy the person’s needs

+3 - Substantial facilitator: 50–95% positive effect; transportation services are available, widely accessible, of good quality, and almost fully satisfy the person’s needs.

+4 - Complete facilitator: 96–100% positive effect; transportation services are available, widely accessible, of high quality, and fully satisfy the person’s needs.

e550 Legal services, systems and policies

Services, systems and policies concerning the legislation and other laws of a country.

e5500 Legal services

Services and programmes aimed at providing the authority of the state as defined in law, such as courts, tribunals and other agencies for hearing and settling civil litigation and criminal trials, attorney representation, services of notaries, mediation, arbitration and correctional or penal facilities, including those who provide these services.

0 - No or very low barrier: 0–4%.

-1 - Mild barrier: 5–24% negative effect; legal services are inconsistently available, but services are of inconsistent quality.
-2 - Moderate barrier: 25–49% negative effect; legal services are sparsely available and services are of inconsistent quality.
-3 - Severe barrier: 50–95% negative effect; legal services are sparsely available and services are of low quality.
-4 - Complete barrier: 96–100% negative effect; legal services are not available.
0 - No or very low facilitator: approximately 0–4%.
+1 - Mild facilitator: 5–24% positive effect; legal services are available, access is variable, but services do not adapt well to the person's needs.
+2 - Moderate facilitator: 25–49% positive effect; legal services are available and widely accessible, but services only partly satisfy the person's needs
+3 - Substantial facilitator: 50–95% positive effect; legal services are available, widely accessible, of good quality, and almost fully satisfy the person's needs.
+4 - Complete facilitator: 96–100% positive effect; legal services are available, widely accessible, of high quality, and fully satisfy the person's needs.

e555 Associations and organizational services, systems and policies
Services, systems and policies relating to groups of people who have joined together in the pursuit of common, noncommercial interests, often with an associated membership structure.
e5550 Associations and organizational services
Services and programmes provided by people who have joined together in the pursuit of common, noncommercial interests with people who have the same interests, where the provision of such services may be tied to membership, such as associations and organizations providing recreation and leisure, sporting, cultural, religious and mutual aid services.

0 - No or very low barrier: 0–4%.
-1 - Mild barrier: 5–24% negative effect; associations and organizational services are inconsistently available, but services are of inconsistent quality.
-2 - Moderate barrier: 25–49% negative effect; associations and organizational services are sparsely available and services are of inconsistent quality.
-3 - Severe barrier: 50–95% negative effect; associations and organizational services are sparsely available and services are of low quality.
-4 - Complete barrier: 96–100% negative effect; associations and organizational services are not available.

0 - No or very low facilitator: 0–4%.
+1 - Mild facilitator: 5–24% positive effect; associations and organizational services are available, access is variable, but services do not adapt well to the person's needs.
+2 - Moderate facilitator: 25–49% positive effect; associations and organizational services are available and widely accessible, but services only partly satisfy the person's needs
+3 - Substantial facilitator: 50–95% positive effect; associations and organizational services are available, widely accessible, of good quality, and almost fully satisfy the person's needs.
+4 - Complete facilitator: 96–100% positive effect; associations and organizational services are available, widely accessible, of high quality, and fully satisfy the person's needs.

e570 Social security services, systems and policies
Services, systems and policies aimed at providing income support to people who, because of age, poverty, unemployment, health condition or disability, require public assistance funded by general tax revenues or contributory schemes.
Exclusion: economic services, systems and policies (e565)
e5700 Social security services
Services and programmes aimed at providing income support to people who, because of age, poverty, unemployment, health condition or disability, require public assistance that is funded either by general tax revenues or contributory schemes, such as services for determining eligibility, delivering or distributing assistance payments for the following types of programmes: social assistance programmes (e.g., noncontributory welfare, poverty or other needs-based compensation), social insurance programmes (e.g., contributory accident or unemployment insurance), and disability and related pension schemes (e.g., income replacement), including those who provide these services.

0 - No or very low barrier: 0–4%.
-1 - Mild barrier: 5–24% negative effect; social security services are inconsistently available, but services are of inconsistent quality.
-2 - Moderate barrier: 25–49% negative effect; social security services are sparsely available, and services are of inconsistent quality.
-3 - Severe barrier: 50–95% negative effect; social security services are sparsely available, and services are of low quality.
-4 - Complete barrier: 96–100% negative effect; social security services are not available.
0 - No or very low facilitator: 0–4%.

+1 - Mild facilitator: 5–24% positive effect; social security services are available, access is variable, but services do not adapt well to the person’s needs.
+2 - Moderate facilitator: 25–49% positive effect; social security services are available and widely accessible, but services only partly satisfy the person’s needs
+3 - Substantial facilitator: 50–95% positive effect; social security services are available, widely accessible, of good quality, and almost fully satisfy the person’s needs.
+4 - Complete facilitator: 96–100% positive effect; social security services are available, widely accessible, of high quality, and fully satisfy the person’s needs.

e575 General social support services, systems and policies
Services, systems and policies aimed at providing support to those requiring assistance in areas such as shopping, housework, transport, self-care and care of others to function more fully in society.
e5750 General social support services
Services and programmes aimed at providing social support to people who, because of age, poverty, unemployment, health condition or disability, require public assistance in the areas of shopping, housework, transport, self-care and care of others to enable them to function more fully in society.

0 - No or very low barrier: 0–4%.
-1 - Mild barrier: 5–24% negative effect; general social support services are inconsistently available, but services are of inconsistent quality.
-2 - Moderate barrier: 25–49% negative effect; general social support services are sparsely available, and services are of inconsistent quality.
-3 - Severe barrier: 50–95% negative effect; general social support services are sparsely available, and services are of low quality.
-4 - Complete barrier: 96–100% negative effect; general social support services are not available.
0 - No or very low facilitator: 0–4%.
+1 - Mild facilitator: 5–24% positive effect; general social support services are available, access is variable, but services do not adapt well to the person’s needs.
+2 - Moderate facilitator: 25–49% positive effect; general social support services are available and widely accessible, but services only partly satisfy the person’s needs
+3 - Substantial facilitator: 50–95% positive effect; general social support services are available, widely accessible, of good quality, and almost fully satisfy the person’s needs.
+4 - Complete facilitator: 96–100% positive effect; general social support services are available, widely accessible, of high quality, and fully satisfy the person’s needs.

e580 Health services, systems and policies

Services, systems and policies for preventing and treating health problems, providing medical rehabilitation and promoting a healthy lifestyle.

e5800 Health services

Services and programmes at a local, community, regional, state or national level aimed at delivering interventions to individuals for their physical, psychological and social wellbeing, such as health promotion and disease prevention services, primary care services, acute care, rehabilitation and long-term care services. Services that are publicly or privately funded, delivered on a short-term, long-term, periodic or onetime basis, in a variety of service settings such as community, home-based, school and work settings, general hospitals, specialty hospitals, clinics, and residential and nonresidential care facilities, including those who provide these services.

0 - No or very low barrier: 0–4%.

-1 - Mild barrier: 5–24% negative effect; health services are inconsistently available, but services are of inconsistent quality.

-2 - Moderate barrier: 25–49% negative effect; health services are sparsely available and services are of inconsistent quality.

-3 - Severe barrier: 50–95% negative effect; health services are sparsely available, and services are of low quality.

-4 - Complete barrier: 96–100% negative effect; health services are not available.

0 - No or very low facilitator: approximately 0–4%.

+1 - Mild facilitator: 5–24% positive effect; health services are available, access is variable, but services do not adapt well to the person's needs.

+2 - Moderate facilitator: 25–49% positive effect; health services are available, and widely accessible, but services only partly satisfy the person's needs

+3 - Substantial facilitator: 50–95% positive effect; health services are available, widely accessible, of good quality, and almost fully satisfy the person's needs.

+4 - Complete facilitator: 96–100% positive effect; health services are available, widely accessible, of high quality, and fully satisfy the person's needs.

e585 Education and training services, systems and policies

Services, systems and policies for the acquisition, maintenance and improvement of knowledge, expertise and vocational or artistic skills. See UNESCO's International Standard Classification of Education (ISCED-1997).

e5850 Education and training services

Services and programmes concerned with education and the acquisition, maintenance and improvement of knowledge, expertise and vocational or artistic skills, such as those provided for different levels of education (e.g., preschool, primary school, secondary school, postsecondary institutions, professional programmes, training and skills programmes, apprenticeships and continuing education), including those who provide these services.

0 - No or very low barrier: 0–4%.

-1 - Mild barrier: 5–24% negative effect; education and training services are inconsistently available, but services are of inconsistent quality.

-2 - Moderate barrier: 25–49% negative effect; education and training services are sparsely available, and services are of inconsistent quality.

-3 - Severe barrier: 50–95% negative effect; education and training services are sparsely available, and services are of low quality.

-4 - Complete barrier: 96–100% negative effect; education and training services are not available.

0 - No or very low facilitator: 0–4%.

+1 - Mild facilitator: 5–24% positive effect; education and training services are available, access is variable, but services do not adapt well to the person's needs.

+2 - Moderate facilitator: 25–49% positive effect; education and training services are available, and widely accessible, but services only partly satisfy the person's needs

+3 - Substantial facilitator: 50–95% positive effect; education and training services are available, widely accessible, of good quality, and almost fully satisfy the person's needs.

+4 - Complete facilitator: 96–100% positive effect; education and training services are available, widely accessible, of high quality, and fully satisfy the person's needs.

e590 Labour and employment services, systems and policies

Services, systems and policies related to finding suitable work for persons who are unemployed or looking for different work, or to support individuals already employed who are seeking promotion.

Exclusion: economic services, systems and policies (e565)

e5900 Labour and employment services

Services and programmes provided by local, regional or national governments or private organizations to find suitable work for persons who are unemployed or looking for different work or to support individuals already employed, such as services of employment search and preparation, reemployment, job placement, outplacement, vocational follow-up, occupational health and safety services, and work environment services (e.g., ergonomics, human resources and personnel management services, labour relations services, professional association services), including those who provide these services.

0 - No or very low barrier: 0–4%.
-1 - Mild barrier: 5–24% negative effect; labour and employment services are inconsistently available, but services are of inconsistent quality.
-2 - Moderate barrier: 25–49% negative effect; labour and employment services are sparsely available, and services are of inconsistent quality.
-3 - Severe barrier: 50–95% negative effect; labour and employment services are sparsely available, and services are of low quality.
-4 - Complete barrier: 96–100% negative effect; labour and employment services are not available.
0 - No or very low facilitator: 0–4%.
+1 - Mild facilitator: 5–24% positive effect; labour and employment services are available, access is variable, but services do not adapt well to the person’s needs.
+2 - Moderate facilitator: 25–49% positive effect; labour and employment services are available, and widely accessible, but services only partly satisfy the person’s needs
+3 - Substantial facilitator: 50–95% positive effect; labour and employment services are available, widely accessible, of good quality, and almost fully satisfy the person’s needs.
+4 - Complete facilitator: 96–100% positive effect; labour and employment services are available, widely accessible, of high quality, and fully satisfy the person’s needs.
