# Supplementary material for: REVISION OF THE BRIEF INTERNATIONAL CLASSIFICATION OF FUNCTIONING, DISABILITY AND HEALTH CORE SET FOR MULTIPLE SCLEROSIS: A STUDY OF THE COMPREHENSIVE ICF CORE SET FOR MULTIPLE SCLEROSIS WITH PARTICIPANTS REFERRED FOR WORK ABILITY ASSESSMENT
Source: J Rehabil Med. 2024 Mar 7;56:19671. doi: 10.2340/jrm.v56.19671 (PMC10938140; doi:10.2340/jrm.v56.19671)
Supplement: REVISION OF THE BRIEF INTERNATIONAL CLASSIFICATION OF FUNCTIONING, DISABILITY AND HEALTH CORE SET FOR MULTIPLE SCLEROSIS: A STUDY OF THE COMPREHENSIVE ICF CORE SET FOR MULTIPLE SCLEROSIS WITH PARTICIPANTS REFERRED FOR WORK ABILITY ASSESSMENT [file JRM-56-19671-s2.pdf]

Supplementary material has been published as submitted. It has not been copyedited, typeset or checked for scientific content by Journal of Rehabilitation Medicine

Table SI. Not evaluated ICF categories

Categories	Description of categories	Reasons for exclusion
b1308	Energy and drive functions, other specified	Other specified functions were not evaluated
b235	Vestibular functions	There was no available data on otholaryngological evaluation for sensory functions of the inner ear related to position, balance and movement
b445	Respiratory muscle functions	There was no available data on functions of the respiratory muscles involved in breathing
b5508	Thermoregulatory functions, other specified –	Other specified functions were not evaluated
b640	Sexual functions	There was no available data on sexual functions related to the sexual act, including the arousal, preparatory, orgasmic and resolution stages. A telephone interview was not considered to be propriate
b710	Mobility of joint functions	There was no available data on functions of the range and ease of movement of a joint
e1108	Products or substances for personal consumption, other specified	Other specified products or substances for personal consumption were not evaluated

Table SII. ICD-10–AM codes of comorbidities and number/proportion of patients with a specific diagnosis

ICD-10-AM code	Diagnosis in text	Number of patients (%)
B18.2	Chronic viral hepatitis C	1 (.7%)
D69.3	Immune thrombocytopenic purpura	1 (.7%)
E03.2	Hypothyroidism due to medicaments and other exogenous substances	1 (.7%)
F06.33	Mood disorder due to known physiological condition with manic features	1 (.7%)
F20	Schizophrenia	1 (.7%)
F33.1	Major depressive disorder, recurrent, moderate	3 (2.0%)
F33.2	Major depressive disorder, recurrent severe without psychotic features	1 (.7%)
G54.1	Lumbosacral plexus disorders	1 (.7%)
G54.4	Lumbosacral root disorders, not elsewhere classified	1 (.7%)
H44.2	Degenerative myopia	1 (.7%)
H47.2	Optic atrophy	4 (2.6%
H54.4	Blindness, one eye	1 (.7%)
I11.0	Hypertensive heart disease with heart failure	2 (1.3%)
I11.9	Hypertensive heart disease without heart failure	17 (11.3%)
I50	Heart failure	2 (1.3%)
L40.0	Psoriasis vulgaris	1 (.7%)
M24.55	Contracture, hip	1 (.7%)
M51.1	Thoracic, thoracolumbar and lumbosacral intervertebral disc disorders with radiculopathy	2 (1.3%)
M51.3	Other thoracic, thoracolumbar and lumbosacral intervertebral disc degeneration	1 (.7%)
S22.03	Fracture of third thoracic vertebra	1 (.7%)
S72.01	Unspecified intracapsular fracture of femur	1 (.7%)

Table SIII. Comprehensive ICF Core Set for Multiple Sclerosis and proportion of patients with impaired body functions.

**Body Functions**	**Proportion of patients with impaired body function**
b114 Orientation functions	20.5%
b126 Temperament and personality functions	74.8%
b1300 Energy level	72.8%
b1301 Motivation	50.7%
b134 Sleep functions	54.3%
b140 Attention functions	57.0%
b144 Memory functions	70.2%
b152 Emotional functions	46.4%
b156 Perceptual functions	4.0%
b164 Higher-level cognitive functions	35.1%
b210 Seeing functions	74.2%
b260 Proprioceptive function	64.2%
b265 Touch function	54.3%
b270 Sensory functions related to temperature and other stimuli	23.8%
b280 Sensation of pain	64.2%
b310 Voice functions	4.0%
b320 Articulation functions	11.9%
b330 Fluency and rhythm of speech functions	46.4%
b455 Exercise tolerance functions	94.0%
b5104 Salivation	18.5%
b5105 Swallowing	40.4%
b525 Defecation functions	52.3%
b5500 Body temperature	68.2%
b620 Urination functions	81.5%
b730 Muscle power functions	92.1%
b735 Muscle tone functions	44.0%
b740 Muscle endurance functions	73.5%

b750 Motor reflex functions	81.5%
b760 Control of voluntary movement functions	88.7%
b7650 Involuntary contractions of muscles	50.3%
b7651 Tremor	37.1%
b770 Gait pattern functions	91.3%
b780 Sensations related to muscles and movement functions	43.0%
**Body Structures**	
s110 Structure of brain	98.7%
s120 Spinal cord and related structures	88.1%
**Activities & Participation**	
d110 Watching	5.3%
d155 Acquiring skills	19.9%
d160 Focusing attention	17.2%
d163 Thinking	6.6%
d166 Reading	20.5%
d170 Writing	36.4%
d175 Solving problems	14.6%
d177 Making decisions	6.6%
d210 Undertaking a single task	5.3%
d220 Undertaking multiple tasks	52.3%
d230 Carrying out daily routine	12.6%
d240 Handling stress and other psychological demands	60.3%
d330 Speaking	4.0%
d350 Conversation	6.6%
d360 Using communication devices and techniques	5.3%
d410 Changing basic body position	49.0%
d415 Maintaining a body position	70.2%
d420 Transferring oneself	34.4%
d430 Lifting and carrying objects	25.8%
d440 Fine hand use	35.8%
d445 Hand and arm use	43.7%
d450 Walking	84.1%
d455 Moving around	94.7%

d460 Moving around in different locations	68.9%	
d465 Moving around using equipment	92.8%	
d470 Using transportation	27.8%	
d475 Driving	58.9%	
d510 Washing oneself	21.9%	
d520 Caring for body parts	25.8%	
d530 Toileting	6.6%	
d540 Dressing	9.9%	
d550 Eating	6.0%	
d560 Drinking	5.3%	
d570 Looking after one’s health	9.3%	
d620 Acquisition of goods and services	43.7%	
d630 Preparing meals	23.2%	
d640 Doing housework	36.4%	
d650 Caring for household objects	39.7%	
d660 Assisting others	12.6%	
d710 Basic interpersonal interactions	6.0%	
d720 Complex interpersonal interactions	21.9%	
d750 Informal social relationships	11.3%	
d760 Family relationships	27.8%	
d770 Intimate relationships	33.1%	
d825 Vocational training	4.7%	
d830 Higher education	10.2%	
d845 Acquiring, keeping and terminating a job	53.6%	
d850 Remunerative employment	70.9%	
d860 Basic economic transactions	13.2%	
d870 Economic self-sufficiency	53.6%	
d910 Community life	32.5%	
d920 Recreation and leisure	64.2%	
d930 Religion and spirituality	3.3%	
**Environmental Factors**	**Barriers**	**Facilitators**
e1101 Drugs	1.3%	75.5%
e115 Products and technology for personal use in daily living	13.2%	10.6%

e120 Products and technology for personal indoor and outdoor mobility and transportation	4.6%	5.3%
e125 Products and technology for communication	0.0%	1.3%
e135 Products and technology for employment	0.0%	0.0%
e150 Design, construction and building products and technology of buildings for public use	3.3%	0.0%
e155 Design, construction and building products and technology of buildings for private use	5.3%	2.7%
e165 Assets	12.6%	2.6%
e2250 Temperature	49.7%	0.0%
e2251 Humidity	23.8%	0.0%
e2253 Precipitation	21.9%	0.7%
e310 Immediate family	10.6%	78.8%
e315 Extended family	0.7%	2.0%
e320 Friends	4.6%	62.9%
e325 Acquaintances, peers, colleagues, neighbours and community members	5.3%	43.0%
e330 People in positions of authority	15.2%	26.5%
e340 Personal care providers and personal assistants	7.3%	2.0%
e355 Health professionals	5.3%	89.4%
e360 Other professionals	7.9%	2.6%
e410 Individual attitudes of immediate family members	4.0%	74.2%
e415 Individual attitudes of extended family members	0.0%	0.0%
e420 Individual attitudes of friends	0.7%	44.4%
e425 Individual attitudes of acquaintances, peers, colleagues, neighbours and community members	2.0%	24.5%
e430 Individual attitudes of people in positions of authority	7.3%	21.2%
e440 Individual attitudes of personal care providers and personal assistants	0.7%	5.3%
e450 Individual attitudes of health professionals	2.0%	77.5%
e460 Societal attitudes	6.0%	81.5%
e515 Architecture and construction services, systems and policies	2.0%	0.7%
e525 Housing services, systems and policies	2.0%	0.7%
e540 Transportation services, systems and policies	7.3%	0.7%
e550 Legal services. systems and policies	0.7%	0.0%

e555 Associations and organizational services. systems and policies	0.7%	9.3%
e570 Social security services, systems and policies	4.0%	75.5%
e575 General social support services, systems and policies	5.3%	11.9%
e580 Health services, systems and policies	2.0%	81.5%
e585 Education and training services, systems and policies	1.3%	2.0%
e590 Labour and employment services, systems and policies	51.7%	47.7%
